# Mitochondrial glutamine synthetase overexpression in tomato source leaves increases assimilate export and fruit yield

**DOI:** 10.1093/plcell/koag181

**Published:** 2026-06-13

**Authors:** José G Vallarino, Laise Rosado-Souza, Youjun Zhang, R George Ratcliffe, Lee J Sweetlove, Sanu Shameer, Alisdair R Fernie

**Affiliations:** Max Planck Institute for Molecular Plant Physiology, Potsdam-Golm, Germany; Faculty of Sciences, University of Málaga, IHSM-UMA-CSIC Campus Teatinos, Málaga, Spain; Max Planck Institute for Molecular Plant Physiology, Potsdam-Golm, Germany; State Key Laboratory of Seed Innovation, Institute of Genetics and Developmental Biology, Chinese Academy of Sciences, Beijing, China; College of Advanced Agricultural Sciences, University of Chinese Academy of Sciences, Beijing, China; Department of Plant Sciences, University of Oxford, Oxford, United Kingdom; Department of Plant Sciences, University of Oxford, Oxford, United Kingdom; Department of Plant Sciences, University of Oxford, Oxford, United Kingdom; School of Biology, Indian Institute of Science Education and Research Thiruvananthapuram, Maruthamala P.O., Kerala, India; Max Planck Institute for Molecular Plant Physiology, Potsdam-Golm, Germany; Center of Plant Systems Biology and Biotechnology, Plovdiv, Bulgaria

## Abstract

To identify potential strategies for increasing the efficiency of tomato leaf metabolism with a focus on the links between nitrogen/carbon metabolism, we explored a diel flux balance analysis (FBA) model of a source leaf in which the metabolic output was varied up to the theoretically achievable maximum. We noticed a potentially interesting switch in the use of glutamine synthetase (GS) isoforms—from the chloroplast isoform to the mitochondrial one—for nitrogen assimilation. To further explore this prediction, we characterized transgenic tomato plants as overexpressing 2 tomato *GS* genes, *GS1* and *GS2*, targeted to mitochondria. Both sets of transgenic plants were characterized as displaying faster growth rate, early flowering, and increased fruit yield. In leaves, metabolomic profiling and enzyme activity analysis pointed out that GS activity in mitochondria plays a role in increasing the intracellular synthesis and subsequent export of sugar. Consistent with these changes, higher sucrose concentration in leaf exudates and reduced activities of enzymes involved in leaf starch synthesis were observed. Moreover, mitochondrial GS activity affected chloroplast redox status in a manner that modulated photorespiration and nitrogen metabolism. The combined data reveal the influence of mitochondrial GS activity on both foliar carbon/nitrogen balance and regulation of source-sink metabolism in tomato plants.

## Introduction

Research on fruit development and ripening in crop species that have agricultural value is essential given that these processes influence yield, quality, and harvesting efficiency (Klee and Giovannoni 2011; Seymour et al. 2013; Kwon et al. 2020; Martín-Pizarro et al. 2021; Ren et al. 2021). Similarly, advances have been made recently in understanding the mechanisms that link metabolism to crop yield (Riedelsheimer et al. 2012a, 2012b; de Abreu et al. 2017), including fruit yield (Schauer et al. 2006, 2008; Do et al. 2010). That said, there is considerable need to better understand metabolic factors that affect fruit yield so they can be targeted to improve breeding strategies or for genetic manipulation. Within this context considerable recent evidence has accrued that highlights the importance of nitrogen (N) metabolism with respect to carbon (C) partitioning rendering metabolites at the interface of C/N metabolism as attractive targets for the manipulation of source-sink balance (Santiago and Tegeder 2016, 2017; Tegeder and Masclaux-Daubresse 2018; Fernie et al. 2020; Lu et al. 2020). That C and N metabolism are strongly intertwined is known at many levels; for example, amino acids are vital to the assembly and operation of the photosynthetic apparatus, while photosynthesis and photorespiration provide energy, reductants, and C skeletons for N reduction and amino acid synthesis. C positively regulates N assimilation and metabolism at transcriptional and post-translational levels; for example, the control of nitrate reductase (NR) expression (Vincentz et al. 1993) as well as its activity by phosphorylation and interaction with inhibitor proteins (Bachmann et al. 1996). That said, legumes with increased N acquisition, source-to-sink transport of organic N, seed development, and Nitrogen Use Efficiency (NUE) suggest that C availability does not always present a hurdle to such improvements (Zhang et al. 2015; Santiago and Tegeder 2016), given that plants are able to adjust their C metabolism and transport in response to N availability and sink strength (Zhang et al. 2015; Santiago and Tegeder 2016). In addition, in *Arabidopsis* or other oilseed plants, enhanced amino acid transport toward source leaves and accompanied improvements in photosynthetic NUE can lead to increased seed C supply and, ultimately, yield, even under N stress conditions (Perchlik and Tegeder 2018). Intriguingly, flexible adjustment in C and N metabolism and allocation may also occur in the reverse situation, at least under high N availability. A recent study in pea shows that enhanced sucrose partitioning from source to sink results in modifications in leaf C and N metabolism as well as amino acid transport to sinks to accommodate the increased assimilate demand of growing seeds (Lu et al. 2020). Indeed, taking such a combined strategy whereby both C and N metabolism are simultaneously altered appears particularly prudent given difficulties in enhancing tomato fruit yield following multi-gene transformation strategies (Vallarino et al. 2020).

Despite the fact that considerable advances and notable successes have been made in engineering plant metabolism (Zhu et al. 2017; Mangel et al. 2019; Vallarino et al. 2020; Hsieh et al. 2021), our ability to rationally manipulate central metabolism that provides constituents for biomass and other outputs remains limited, largely because of the highly interconnected nature of the central metabolic network. It is therefore essential to approach metabolism from a systematic perspective when developing hypotheses for metabolic engineering (Sweetlove et al. 2017) and computational models are central to this endeavor (Küken and Nikoloski 2019; Clark et al. 2020). For large networks like central metabolism, flux balance analysis (FBA) has emerged as a robust approach to make accurate predictions of fluxes and as a useful in-silico tool to explore network capabilities and identify engineering strategies (Kruger and Ratcliffe 2015). A constraint-based modeling approach is increasingly being used to model metabolism in many plant tissues (Grafahrend-Belau et al. 2013; Colombié et al. 2015; Yuan et al. 2016; diCenzo et al. 2020; Shameer et al. 2020) and in specific cell types (Robaina-Estévez et al. 2017; Tan and Cheung 2020).

In the current study, we used a diel FBA model (Cheung et al. 2014) of metabolism of a tomato source leaf to explore potential flux distributions in the metabolic network as the system approached its theoretical-maximum in terms of efficiency (in this case the amount of sugars and amino acids that could be exported from the source leaf for a given rate of photosynthetic C assimilation). One of the interesting changes we noted at the intersection of N and C metabolism involved glutamine synthetase (GS), with the model suggesting an advantage in boosting the capacity of mitochondrial GS. In plants, there are 2 GS isoforms, a cytosolic form (GS1) and a plastidic form (GS2) (Bernard and Habash 2009), that catalyze the ATP-dependent synthesis of glutamine (Gln) from NH_4_^+^ and glutamate (Glu). Taira et al. (2004) in Arabidopsis has pointed out that GS2 form functions in both leaf mitochondria and chloroplast to facilitate NH_4_^+^ recovery during photorespiration. Moreover, the SUBA4 database suggested that there is also proteomic evidence for the presence of GS2 in Arabidopsis mitochondria (Hooper et al. 2017).

In this work, we generated 2 sets of transgenic tomato lines (cv MoneyMaker) exhibiting either increasing expression of *GS1* or *GS2* in mitochondria under the control of leaf- and mesophyll-specific promoter, ribulose-bisphosphate carboxylase (RbcS), which confers leaf-mesophyll specificity, respectively. We carried out an extensive phenotypic and molecular analysis of these lines encompassing analysis of primary metabolite levels as well as expression profiles and cellular fluxes of these lines. The integrated analysis of these comprehensive sets of data allows us to discuss these data with respect to current models of the integration of N and C metabolism during plant growth and fruit ripening and development. This approach clearly represents a novel strategy for agriculture as highlighting the power of combining computational modeling with experimental research within approaches to metabolically engineer organ growth.

## Results

### In silico exploration of efficiency of the tomato source leaf metabolic network

To identify points at the intersection of N and C metabolism that could be potential targets for improving the overall efficiency of leaf metabolism, we constructed a diel stoichiometric model of central metabolism in a tomato source leaf and predicted fluxes in the network using parsimonious flux balance analysis (pFBA). The model was required to produce sugars and amino acids in specified relative proportions as observed in tomato phloem sap (Walker and Ho 1977; Valle et al. 1998). Non–growth-associated maintenance costs were also accounted for according to the incident light intensity (Töpfer et al. 2020). Further details of model set-up can be found in the Methods section, and a full list of constraints applied to the model is provided (Table S1). Our strategy to identify fluxes of interest was to perform a constraint scan, varying the output of the model (export of sugars and amino acids to the phloem) from the maximum predicted by pFBA. To do this, the output reaction flux was reduced from the maximum in 2% steps, each time re-running the model and storing the flux predictions. The sets of flux predictions obtained were plotted against model output to identify reactions that show marked changes in behavior as the model approached the theoretical maximum output. Flux-variability ranges were computed for reactions of interest to account for the variability of fluxes within the model solution space. A full dataset of the flux distributions from this process is provided in Data Set S1. Of the reactions involved in N assimilation in the leaf, we noticed a potentially interesting behavior with the glutamine synthetase (GS) reaction (Fig. S1). The output of the model was positively correlated with overall GS reaction flux, which is not surprising as the output includes amino acids whose synthesis requires the assimilation of nitrate in the leaf. But additionally, there was a marked switch in the choice of GS isoform used as the model reached approximately 90% of the theoretical maximum. Up to that point, N was assimilated exclusively using the chloroplast GS. Beyond that point, the contribution of the chloroplast GS began to decline, and the mitochondrial GS took over. Very close to the theoretical maximum (∼97%) a small flux in cytosolic GS was predicted, although flux variability analysis indicated that this small flux could be entirely taken up by additional mitochondrial GS with no detriment to the output (Fig. S1). These predictions suggest that there may be an overall efficiency gain by introducing a functional GS into leaf mitochondria.

### Mitochondrial overexpression of *GS1* and *GS2* in tomato (cv. MoneyMaker) leaves

**Figure 1:**
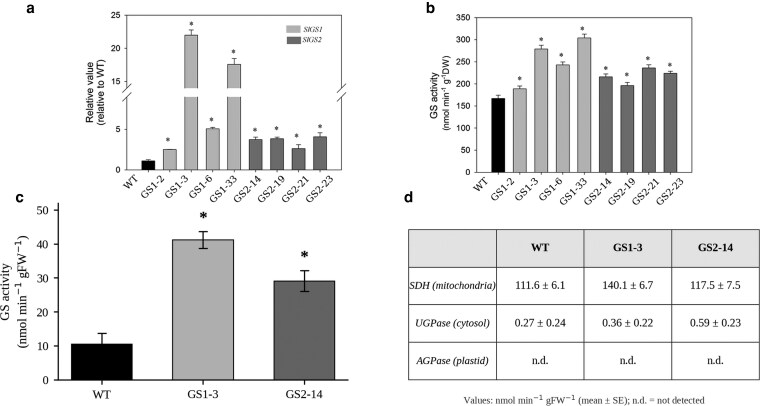
Characterization of transgenic lines overexpressing mitochondrially targeted GS1 and GS2. **a)** Expression of tomato *SlGS1* and *SlGS2* genes by qRT-PCR in source leaves. **b)** Total GS activity in source leaves (enzyme activity was normalized to dry weight). For A and B, data were obtained from the third fully expanded leaf from the apex of 6-wk-old plants. Leaves were harvested at midday. Values are means from 5 replicates ± standard error. Statistically significant differences by Student *t* test are indicated by asterisk (*P* < 0.05). **c)** GS activity measured in purified mitochondrial fractions isolated from leaves of wild-type, GS1-3, and GS2-14 plants (nmol min^−1^ gFW^−1^). Values are means from 5 replicates ± standard error. Asterisks indicate statistically significant differences compared with wild type (*P* < 0.01). **d)** Marker enzyme activities in the same purified mitochondrial fractions: succinate dehydrogenase (SDH, mitochondrial marker), UDP-glucose pyrophosphorylase (UGPase, cytosolic marker), and ADP-glucose pyrophosphorylase (AGPase, plastidial marker). Values are means ± SE (nmol min^−1^ gFW^−1^); n.d., not detected.

Full-length cDNAs encoding *GS1* (*Solyc04g014510*) and *GS2* (*Solyc01g080280*) were isolated by a PCR-based approach. A 1,071-bp fragment of the cDNA encoding GS1 was cloned in the sense orientation into the transformation vector pK2GW7 under the control of the leaf mesophyll-specific promoter (RbcS) and in frame with the mitochondrial transit peptide COX4ts (which results in a mitochondrial localization of the encoded protein) (Nelson et al. 2007). Similarly, a 1,316-bp fragment of the cDNA encoding GS2 was cloned in the sense orientation behind the RbcS promoter and the COX4ts mitochondrial transit peptide. Using an established *Agrobacterium tumefaciens*–mediated gene transformation protocol, we were able to generate 10 and 14 independent transgenic tomato lines harboring *RbcS-COX4ts-GS1* and *RbcS-COX4ts-GS2* constructs, respectively. The expression levels of *GS1* and *GS2* were subsequently analyzed in the leaves. Expression was detected in all transgenic lines and the untransformed wild type (control; data not shown). Four lines per construct were chosen: GS1-2, GS1-6, GS1-33, and GS1-3 presenting low, medium, and high overexpression (RbcS-COX4ts-GS1 lines) and GS2-14, GS2-19, GS2-21, and GS2-23 (RbcS-COX4ts-GS2 lines), which displayed more homogeneous overexpression between lines (Fig. 1a). Analysis of GS activity revealed that these lines displayed significant activity increases between 1.2- and 1.8-fold in leaves (Fig. 1b).

Subcellular targeting was tested by transient expression experiments in which both coding regions (CDS) of GS1 or GS2 and the CDS fused to the mitochondrial transit peptide COX4ts were linked with fluorescent GFP. The cytosol, plastid, and mitochondria mCherry constructs were used as the marker of these organelles. The transient agro-infiltration method developed by Zhang et al. (2020) was used to infiltrate *Nicotiana benthamiana* leaves; after 3 d, fluorescence was examined by confocal microscopy. As described in previous works, the native GS1 was largely subcellularly localized to the cytosol, while GS2 was present in both the cytosol and plastid. However, when the mitochondrial signal peptide was fused to either GS1 or GS2, their localization was only in correspondence with the mitochondrial marker (Fig. S2a–d). To assess if this was also the case in the transgenic lines, we isolated mitochondria from the wild type and the strongest overexpressing line of both GS1 (line GS1-3) and GS2 (line GS2-14) and determined the activities of GS as well as the marker enzymes UDP-glucose pyrophosphorylase (UGPase [cytosol]) and ADP-glucose pyrophosphorylase (AGPase [chloroplast]) as well as the additional mitochondrial enzyme succinate dehydrogenase, SDH, to account for the loss of mitochondria during the isolation process. Marker enzyme activities confirmed minimal cytosolic and plastidic contamination (UGPase and AGPase latencies below 3% in all cases; Fig. 1d), while GS activity was significantly enriched in purified mitochondria of the transgenics (10.5 ± 3.2, 41.2 ± 2.5, and 29.1 ± 3.1 nmol min^−1^ gFW^−1^ for wild type, GS1-3, and GS2-14, respectively; Fig. 1c; Table S3). Indeed, although marginally higher in the mitochondria, the increase in mitochondrial GS activity was not significantly different from the total cellular increase of GS activity measures in this harvest (compare 30.7 and 18.6 nmol min^−1^ gFW^−1^ as mitochondrial GS activity increases in lines GS1-3 and GS2-14 with cellular GS activity increases of 29.1 and 17.2 nmol min^−1^ gFW^−1^ in the same lines, respectively). These data alongside the GFP localization data thus provide compelling evidence that the transgene product was successfully and completely targeted to mitochondria.

### Cytosolic and plastidial GS overexpression does not alter plant growth or fruit yield

To determine whether the observed phenotypic effects were specifically dependent on mitochondrial localization of GS or could result from a general increase in GS activity in any subcellular compartment, we generated additional control lines. Full-length cDNAs encoding GS1 and GS2 were cloned under the RbcS promoter but without the COX4ts mitochondrial transit peptide, directing GS1 to its native cytosolic compartment (cytGS1 lines) and GS2 to the plastid (plGS2 lines). Five independent lines per construct were characterized (Figs 3 and 4). The cytGS1 lines exhibited a wide range of transgene expressions, with total GS activity increasing ranging from 50% to 6,000% over wild type (1.5- to 60-fold; Fig. S3d). The plGS2 lines displayed more moderate increases in total GS activity, ranging from 140% to 450% over wild type (2.4- to 5.5-fold; Fig. S3e). Despite these substantial increases in GS enzyme activity, none of the cytGS1 or plGS2 lines displayed significant differences compared with wild-type plants in vegetative growth parameters (plant height, stem dry weight, root dry weight), flowering time, fruit yield, fruit quality (Brix), or fruit diameter (Figs. S3f,g and S4a–f). Fruit diameter was consistently lower than in wild-type plants across both cytGS1 and plGS2 lines (∼10% reduction on average); however, this trend did not reach statistical significance with the number of replicates available and was not accompanied by a reduction in total fruit yield. These results demonstrate that increased GS activity alone is insufficient to produce the growth and yield phenotypes observed in the mitochondrially targeted lines and that the mitochondrial localization of GS is specifically required, consistent with the predictions of the diel FBA model.

### Leaf-specific overexpression of mitochondrially targeted *SlGS1* or *SlGS2* results in increased plant biomass and fruit yield

**Figure 2:**
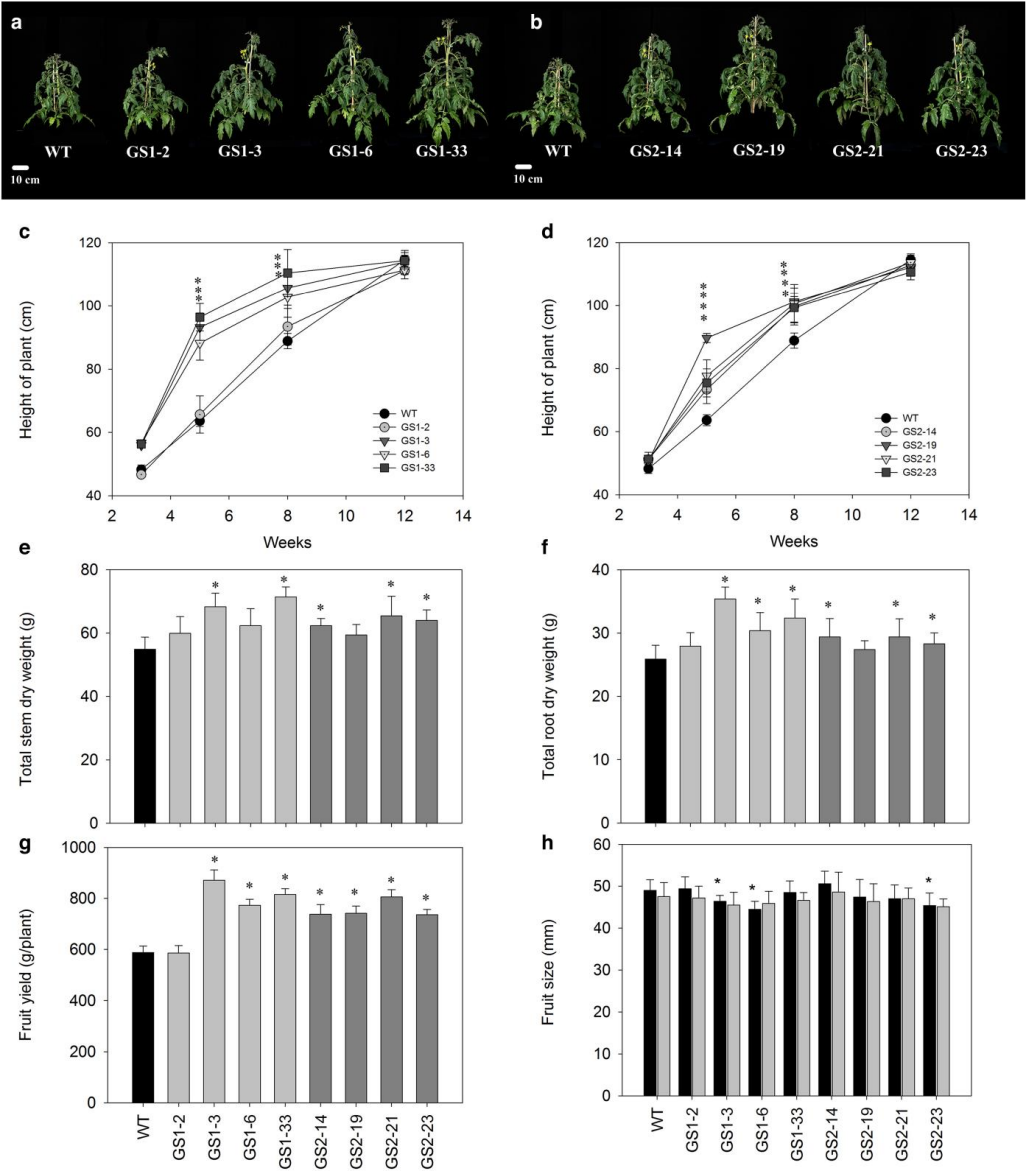
Phenotypic characterization of the *RbcS–GS1* and *RbcS–GS2* lines. **a, b)** Pictures of 5-wk-old plants showed faster growth and early flowering. **c, d)** Plant height along plant development. **e, f)** Total stem and root dry weight in plants of 16 wk, respectively. **g)** Total fruit yield. **h)** Total fruit size. Values are shown as means ± SE of 5 individual plants. Asterisks indicate statistically significant differences determined by Student *t* test (*P* < 0.05). In panel H, black and grey bars represent fruit width and fruit height, respectively. Data corresponds to the spring growing season. Results from the autumn season are presented in Figure S6.

To assess the effect of mitochondrially targeted *GS1* and *GS2* overexpression across the growth and development periods of tomato plants, a detailed phenotypic analysis was performed. Interestingly, when transgenic plants were grown in greenhouse side-by-side with untransformed controls, we observed a clear difference in growth at the beginning of development. Both sets of transgenic lines, *RbcS-COX4ts-GS1* and *RbcS-COX4ts-GS2*, displayed a significantly faster growth rate, with *RbcS-COX4ts-GS1* lines being slightly taller than *RbcS-COX4ts-GS2* plants, but both being taller than the untransformed controls (Fig. 2a–d).

Both sets of transformants consistently exhibited early flowering (Fig. S5a). This was generally accompanied by an earlier appearance of the first red fruit (Fig. S5b). The exception was line GS2-23, which displayed significantly earlier flowering but did not show a reduction in the time from anthesis to the first ripe fruit. This is consistent with the expected variability among independent transgenic lines and does not affect the overall conclusion, since GS2-23 did exhibit increased fruit yield (Fig. 2g) and other phenotypic traits comparable with the remaining transgenic lines (Fig. 2, e and f). The total period of fruit ripening was unaltered in all lines.

Despite the observed difference in early growth rate, after 12 wk the transgenic lines showed no differences from control in plant height. However, at this plant developmental stage, some lines of the transgenic plants displayed an increase in total stem dry weight (GS1-3, GS1-33, GS2-14, GS2-21, and GS2-23) and increased total root dry weight (GS1-3, GS1-6, GS1-33, GS2-14, GS2-21, GS2-23) (Fig. 2e and f). Interestingly, *RbcS-COX4ts-GS1* and *RbcS-COX4ts-GS2* lines were characterized by a significant increase in fruit yield (Fig. 2g), which was a consequence of an increase in number of fruits rather than fruit size or weight, which were invariant across the lines (Fig. 2h). Finally, mature fruits from both sets of transformants showed no significant changes in soluble solid content (^o^Brix; 3.2 ± 0.2; 3.3 ± 0.1; 3.3 ± 0.1; 3.2 ± 0.3; 3.1 ± 0.2; 3.1 ± 0.2; 3.1 ± 0.1; 3.2 ± 0.2; 3.3 ± 0.2; 3.0 ± 0.2 for control, GS1-2, GS1-3, GS1-6, GS1-33, and GS2-14, GS2-19, GS2-21, GS2-23, respectively); however, a reduction in acidity was observed in the mature fruits of the *RbcS-COX4ts-GS1*. The reproducibility of the results was checked by growing the transgenic plants in 2 seasons—spring and autumn. With the results from the autumn season being highly comparable with those presented here (Fig. S6).

### Mitochondrial overexpression of *GS1* and *GS2* does not impact photosynthesis

Given the observed alteration in plant growth, we next evaluated gas exchange parameters in 5-wk-old plants at different photon flux densities (PFD) (Fig. S7). Interestingly, we did not observe significant changes in net photosynthesis (*A*) or stomatal conductance (*g_s_*) (Fig. S7a–d); however, we found a slight decrease of maximum PSII photochemical efficiency (F_v_/F_m_) in lines GS1-6 and GS2-23 (Fig. S7f). In view of these changes, we analyzed the electron transport rate (ETR) at different irradiances, since ETR is associated with the flux of electrons passing through PSII in photosynthesis (Tezara et al. 2003). Transgenic lines were characterized by unaltered ETR values measured at low (100 µmol m^−2^) and high (700 µmol m^−2^) irradiances (Fig. S7e). These combined results suggest that overexpression of mitochondrially targeted *GS1* and *GS2* did not greatly influence the photosynthetic process.

### The effect of overexpression of mitochondrially targeted *GS1* or *GS2* on leaf starch and pigments biosynthesis and plastidial redox status

To understand further the impact of overexpressing mitochondrially targeted *GS1* and *GS2* in leaves, we examined starch and pigments levels in fully expanded leaves. Interestingly, we found a reduction of between 25% and 44% in starch levels (with only line GS1-2 exhibiting invariant levels of starch) (Table 1). Additionally, when we measured pigment levels in leaves, we observed a significant increase of chlorophyll a (GS1-3, GS1-33, GS2-14, and GS2-19) and chlorophyll b (GS1-3, GS2-14, and GS2-23; Table 2). Notably, this rise in pigment content did not translate into higher photosynthetic rates on a leaf area basis (Fig. S7), indicating that the increased chlorophyll pool is not rate-limiting for CO_2_ assimilation under the conditions assayed. Finally, by analyzing pyrimidine nucleotide levels, we noted a significant increase in the levels of the reduced pyridine nucleotide NADPH (except in line GS1-2), with an increase in the deduced NADPH/NADP^+^ ratio in the transgenics. However, no changes were noticed in the other pyridine nucleotides, except for the slight decrease in NAD^+^ levels in GS1-3 and GS2-21 lines (Table 1).

**Table 1.** Starch and pyridine nucleotide levels in *RbcS–COX4ts-GS1* and *RbcS–COX4ts-GS2* lines.

Parameters	Wild-type	GS1-2	GS1-3	GS1-6	GS1-33	GS2-14	GS2-19	GS2-21	GS2-23
	µmol/g DW								
Starch	7.14 ± 0.76	6.62 ± 1.04	**4.17** ± **0.86**	**5.17** ± **0.94**	**4.01** ± **1.14**	**5.06** ± **0.75**	**5.55** ± **0.83**	**5.15** ± **0.93**	**4.75** ± **1.03**
NAD^+^	21.50 ± 0.41	20.78 ± 0.35	19.86 ± 0.59	22.16 ± 0.38	21.04 ± 0.30	22.06 ± 0.36	21.89 ± 0.31	**20.01** ± **0.29**	22.78 ± 0.39
NADH	34.76 ± 1.16	33.67 ± 1.07	**30.26** ± **0.96**	32.17 ± 1.11	31.78 ± 1.28	32.67 ± 0.99	31.76 ± 1.39	33.77 ± 1.36	34.03 ± 0.98
NADP^+^	13.06 ± 0.30	12.83 ± 0.27	13.87 ± 0.31	12.69 ± 0.26	13.45 ± 0.33	11.97 ± 0.46	12.03 ± 0.37	13.87 ± 0.33	12.98 ± 0.40
NADPH	5.33 ± 0.25	5.82 ± 0.29	**7.89** ± **0.26**	**7.03** ± **0.18**	**6.78** ± **0.17**	**6.87** ± **0.26**	**6.27** ± **0.19**	**7.02** ± **0.24**	**6.85** ± **0.22**
NADH/NAD^+^	1.86 ± 0.10	1.73 ± 0.07	1.80 ± 0.06	1.58 ± 0.11	**1.56** ± **0.08**	1.59 ± 0.15	**1.53** ± **0.11**	1.74 ± 0.14	1.80 ± 0.10
NADPH/NADP^+^	0.41 ± 0.04	0.47 ± 0.06	**0.62** ± **0.07**	**0.54** ± **0.06**	**0.53** ± **0.05**	**0.53** ± **0.05**	**0.50** ± **0.03**	**0.56** ± **0.04**	**0.54** ± **0.06**

Leaves were harvested at midday. Values are means from 5 biological replicates ± standard error. Statistically significant differences by Student *t* test are indicated by boldface (*P* < 0.05). Abbreviation: DW, Dry weight.

**Table 2.** Pigment contents in RbcS–COX4ts-GS1 and RbcS–COX4ts-GS2 lines.

Pigments	Wild-type	GS1-2	GS1-3	GS1-6	GS1-33	GS2-14	GS2-19	GS2-21	GS2-23
	µmol g^−1^ DW								
Chlorophyll a	1.55 ± 0.05	1.57 ± 0.07	**1.75** ± **0.04**	1.60 ± 0.05	**1.68** ± **0.03**	1.66 ± 0.06	**1.64** ± **0.04**	1.57 ± 0.06	1.59 ± 0.05
Chlorophyll b	0.54 ± 0.04	0.59 ± 0.03	**0.68** ± **0.03**	0.58 ± 0.05	0.70 ± 0.03	0.62 ± 0.03	0.59 ± 0.04	0.57 ± 0.05	**0.63** ± **0.03**
ß-Carotene	0.034 ± 0.005	0.029 ± 0.006	0.032 ± 0.005	0.030 ± 0.004	0.028 ± 0.005	0.031 ± 0.004	0.033 ± 0.005	0.027 ± 0.005	0.035 ± 0.004

Data is presented as means ± SE of 5 biological determinations. Values that are significantly different by Student *t* test are indicated by boldface (*P* < 0.05). Abbreviation: DW, Dry weight.

### Metabolic alteration in leaves and their exudates in the transgenic lines and their wild-type control

**Figure 3:**
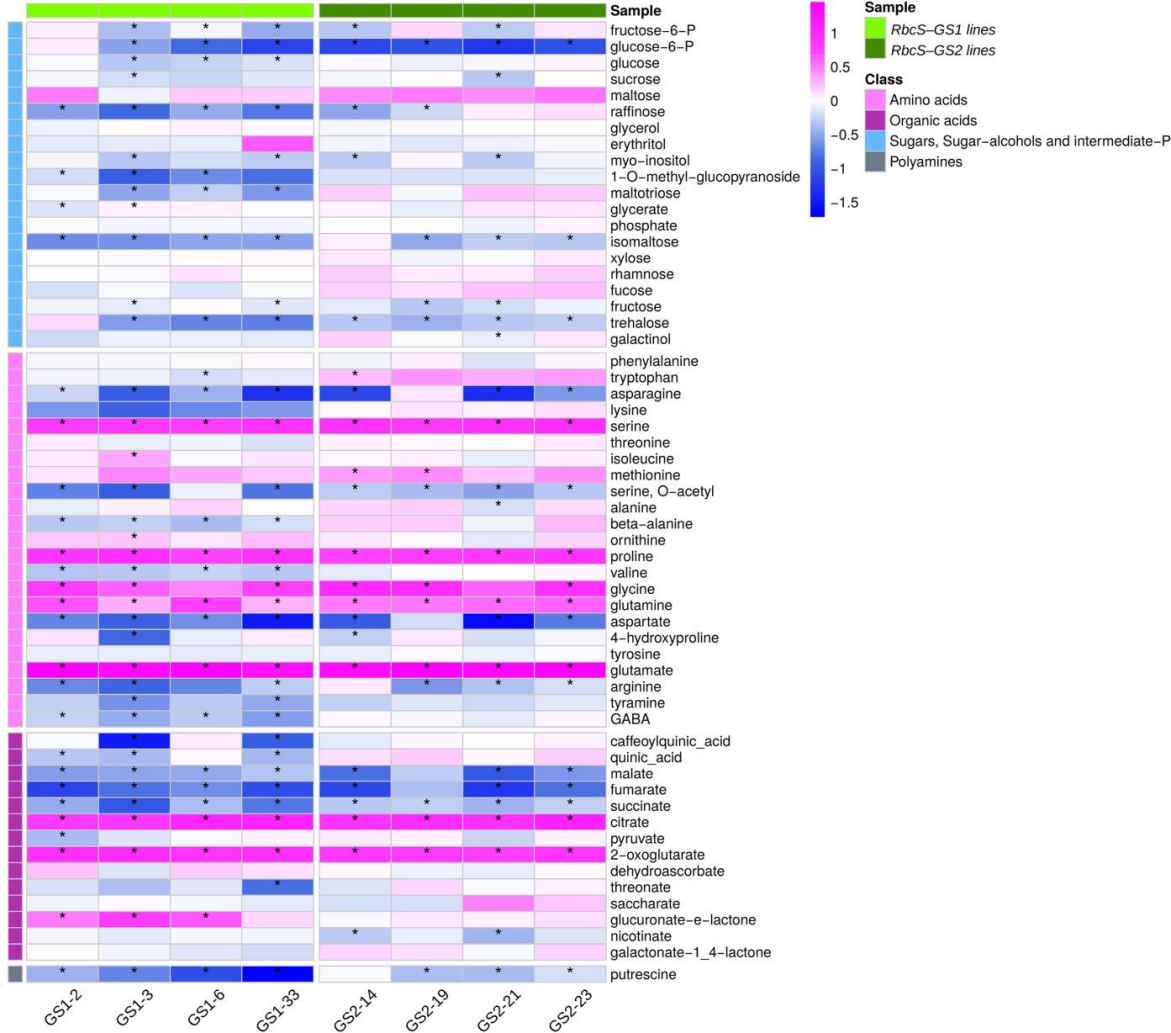
Heatmap of leaf primary metabolites in *RbcS–GS1* and *RbcS–GS2* lines. Data was normalized to dry weight and presented as ratio between transgenic plants and wild type. Asterisks indicate statistically significant differences determined by Student *t* test (*P* < 0.05). The colors indicate the proportional content of each metabolite intensity, which has been log2-transformed and mean-centered. Leaves were harvested at midday from 6-wk-old plants.

Leaves harvested in the middle of the day were analyzed using an established GC-MS–based metabolite profiling protocol (Fernie et al. 2004) (Fig. 3 and Data Set S2). The transgenic lines from both constructs displayed similar changes being characterized by decreases in the main hexoses, glucose (lines GS1-3, GS1-6, and GS1-33) and fructose (lines GS1-3, GS1-33, GS2-19, GS2-21,) as well as in their phosphorylated derivatives, glucose-6-phosphate and fructose-6-phosphate. Similar changes were displayed by the glucose oxidation products, raffinose (all *RbcS-COX4ts-GS1* lines and GS2-14, GS2-19 lines), and by sucrose (lines GS1-3 and GS2-21). Similarly decreases in trehalose (lines GS1-3, GS1-6, GS1-33, and all *RbcS-COX4ts-GS2* lines), isomaltose (all *RbcS-COX4ts-GS1* lines and GS2-19, GS2-21, GS2-23 lines), 1-*O*-methyl-glucopyranoside (lines GS1-2, GS1-3, GS1-6), and *myo*-inositol (lines GS1-3, GS1-33, GS2-14, GS2-21) were observed. Regarding the organic acids, decreased levels of several tricarboxylic acid cycle intermediates, namely succinate (all lines), fumarate (all *RbcS-COX4ts-GS1* lines and GS2-14, GS2-21, GS2-23 lines), and malate (all *RbcS-COX4ts-GS1* lines and GS2-14, GS2-21, GS2-23 lines), were observed, whereas citrate and 2-oxoglutarate levels increased in all transgenic lines. In addition, significant decreases were also observed in caffeoylquinic acid (lines GS1-3 and GS1-33) and quinic acid (lines GS1-2, GS1-3, and GS1-33). Interestingly, some amino acids detected here displayed contrasting behavior, including asparagine (all *RbcS-COX4ts-GS1* lines and GS2-14, GS2-21, GS2-23 lines), β-alanine (all *RbcS-COX4ts-GS1* lines), valine (all *RbcSCOX4ts-GS1* lines), aspartate (all *RbcS-COX4ts-GS1* lines and GS2-14, GS2-21, GS2-23 lines), arginine (lines GS1-2, GS1-3, GS1-33, GS2-19, GS2-21, GS2-23), *O-*acetyl-serine (GS1-2, GS1-3, GS1-33 lines and all *RbcS-COX4ts-GS2* lines), 4-hydroxyproline (lines GS1-3 and GS2-14), and GABA (all *RbcS-COX4ts-GS1* lines), which all decreased in the transgenic lines. By contrast, serine (all lines), glycine (lines GS1-2, GS1-3, GS1-33, GS2-14, GS2-19, GS2-23), glutamine (all lines), proline (all lines), and glutamate (all lines) increased. Moreover, the closely related polyamine putrescine was decreased in all *RbcS-COX4ts-GS1* lines as well as lines GS2-19, GS2-21, and GS2-23. Taken together, these metabolic changes indicate a dramatic shift in primary metabolism with alterations in the levels of sugars, organic acids, and also amino acids.

We next evaluated metabolite levels in phloem exudates of transgenic and control plants. Here, we used authentic standards to perform absolute quantification of metabolite amount (Data Set S3). Phloem exudates from all transgenic lines were characterized by a large increase in the major sugars, glucose, fructose, and sucrose (up to 6.5-fold). Changes were also apparent in glutamate, which decreased in the phloem exudates from all transgenic lines and GABA levels in the exudates from linea GS1-3, GS1-33, GS2-14, and GS2-21.

### Effects of overexpression of mitochondrially targeted *GS1* or *GS2* on enzyme activities concerned with nitrogen assimilation and other aspects of primary metabolism

The aforementioned results indicate that plants overexpressing *GS1* and *GS2* targeted to the mitochondria exhibit reduced starch biosynthesis in source leaves. The plastidic phosphoglucomutase (PGM) and ADP-glucose pyrophosphorylase (AGPase) activities have been described as key enzymes for starch biosynthesis and its regulation (Caspar et al. 1985; Hanson and Mchale 1988; Lin et al. 1988; MacRae and Lunn 2006). In addition, it was previously demonstrated in potato that phospho*enol*pyruvate carboxylase (PEPC) activity also plays an important role in starch metabolism (Rademacher et al. 2002). For this reason, we analyzed the PGM, PEPC, and AGPase activities in leaves of the transgenic and control lines. This analysis revealed that both the *RbcS-COX4ts-GS1* and the *RbcS-COX4ts-GS2* lines displayed significant decreases in PGM and AGPase enzyme activities, whereas PEPC activity was invariant in the transformants (Table 3). In addition, we observed an increase in total and initial activities of NADP-malate dehydrogenase (NADP-MDH) of the chloroplast. These data, when taken together, suggest an altered C metabolism in chloroplast. By contrast, in considering the tricarboxylic acid (TCA) cycle, there was a lack of consistent changes in NAD-MDH and fumarase activities (Table 3).

**Table 3.** Enzymatic activities in RbcS–COX4ts-GS1 and RbcS–COX4ts-GS2 lines.

Enzymes	Wild-type	GS1-2	GS1-3	GS1-6	GS1-33	GS2-14	GS2-19	GS2-21	GS2-23
	nmol min^−1^ g^−1^ DW								
PGM	1,868 ± 126	1,768 ± 156	**1,257** ± **117**	**1,397** ± **137**	**1,308** ± **120**	1,598 ± 129	**1,497** ± **110**	**1,427** ± **147**	**1,528** ± **132**
AGPase PEPC	1,468 ± 86 176 ± 12.6	1,387 ± 69 187 ± 9.3	**1,186** **±** **65** 160 ± 8.8	**1,287** **±** **76** 164 ± 10.3	**1,157** **±** **59** 172 ± 12.1	**1,298** **±** **79** 164 ± 8.4	**1,305** **±** **69** 183 ± 10.4	**1,228** **±** **72** 170 ± 7.6	**1,176** **±** **63** 193 ± 11.5
NADP-MDH total	77.4 ± 5.2	83.4 ± 7.2	**104.3** ± **6.4**	**96.3** ± **6.3**	**117.3** ± **7.5**	**88.2** ± **5.3**	**92.4** ± **6.4**	**93.2** ± **6.3**	**102.3** ± **4.7**
NADP-MDH initial	28.3 ± 2.7	26.3 ± 1.8	**37.7** ± **2.1**	**34.2** ± **1.8**	**41.4** ± **3.2**	**33.7** ± **1.7**	**36.9** ± **2.4**	**39.3** ± **1.3**	**34.6** ± **1.8**
NADP-MDH activation stage	11.7 ± 1.4	10.4 ± 1.7	11.3 ± 1.4	10.2 ± 2.1	9.8 ± 2.1	10.3 ± 1.8	11.3 ± 1.6	12.3 ± 2.4	12.1 ± 1.7
NAD-MDH (mmol min^−1^ g^−1^ DW)	18.3 ± 2.7	16.5 ± 3.9	17.2 ± 2.6	18.5 ± 3.2	**22.3** ± **3.2**	19.4 ± 2.6	18.9 ± 3.5	**20.4** ± **1.7**	16.4 ± 3.5
Fumarase	1,075 ± 82	1,159 ± 92	1,046 ± 103	1,143 ± 79	1,064 ± 72	1,173 ± 93	1,032 ± 103	1,064 ± 94	1,052 ± 83
NR	11.6 ± 4.6	15.8 ± 3.5	**21.4** ± **4.1**	**18.3** ± **3.2**	**17.4** ± **3.1**	**18.3** ± **3.3**	**19.3** ± **4.2**	**20.2** ± **3.1**	**17.3** ± **4.6**
NiR (U min^−1^ g^−1^ DW)	2.1 ± 0.6	1.9 ± 0.8	**1.3** ± **0.4**	**1.1** ± **0.5**	**0.9** ± **0.2**	**1.6** ± **0.4**	**1.3** ± **0.2**	**1.5** ± **0.4**	**1.3** ± **0.2**
GDH	26.4 ± 2.1	**32.4** ± **1.5**	**42.3** ± **2.5**	**39.3** ± **2.1**	**44.5** ± **3.2**	**32.5** ± **1.8**	**29.4** ± **2.6**	**31.4** ± **3.1**	**28.6** ± **1.6**
GOGAT	5.3 ± 1.3	4.7 ± 1.4	**3.2** ± **0.8**	**3.8** ± **0.8**	**2.4** ± **0.9**	4.2 ± 0.7	**3.3** ± **0.8**	**3.6** ± **1.1**	**4.0** ± **0.8**

Leaves were harvested at midday. Values are means from 5 biological replicates ± standard error. Statistically significant differences by Student *t* tests are indicated by boldface (*P* < 0.05). Phosphoglucomutase (PGM), ADP-glucose pyrophosphorylase (AGPase), nitrate reductase (NR), nitrite reductase (NiR), glutamate dehydrogenase (GDH), glutamate synthase (GOGAT). Abbreviation: DW, Dry weight.

Given the amino acid profile obtained and shown in Fig. 3, we examined if leaf-specific mitochondrial GS expression affected N metabolism at the level of enzyme activities. We evaluated the activities of the main N assimilation-related enzymes. Interestingly, the nitrate reductase (NR) activity was increased in transgenic lines, whereas the nitrite reductase (NiR) decreased. Moreover, glutamate dehydrogenase (GDH) activity was significantly higher in all transgenic lines compared with the control; however, the glutamate synthase (GOGAT) activity of the transgenic lines was reduced compared with that observed in the control leaves (Table 3). These results suggest considerable shift in N metabolism in the transgenic leaves. Taking this observation into account, alongside the altered glycine/serine levels displayed by the transgenic lines (Fig. 3), we explored the photorespiratory interconnection between N and C metabolism in these lines. In mitochondria, one of the key reactions of photorespiration is catalyzed by the glycine decarboxylase complex (GDC), which catalyzes the reaction converting 2 molecules of glycine in 1 molecule of serine generating CO_2_ and NH_4_^+^ (Bauwe et al. 2010). GDC is a hetero-tetramer formed by 4 enzymes (P-, T-, H-, and L-) and their combined activity has been described as the major control point for photorespiratory flux being subject to multi-level regulation (Timm 2020). The L-protein (lipoamide dehydrogenase) was selected as indicator because, as a flavoprotein whose activity depends on the mitochondrial NAD+/NADH ratio, it is the GDC subunit most directly connected to the mitochondrial redox state and therefore the component most likely to be transcriptionally responsive to the metabolic changes induced by mitochondrial GS overexpression (Timm 2020). Interestingly, the expression level of the GDC L-protein was significantly higher in all transgenic lines compared with the wild type (Fig. S8).

### Metabolic alterations during development and ripening of the RbcS-COX4ts-GS1 and RbcS-COX4ts-GS2 fruits

**Figure 4:**
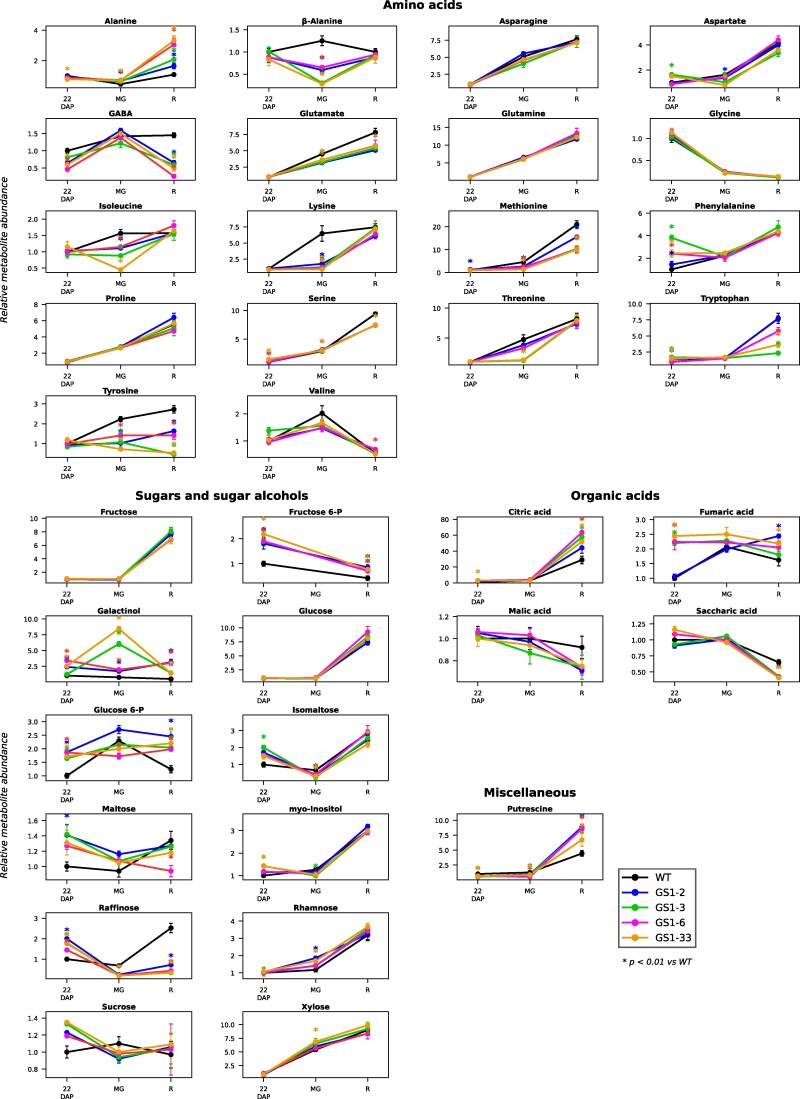
Primary metabolites measured in tomato fruits of wild-type and RbcS–GS1 lines at 3 developmental and ripening stages (22 d after pollination (DAP), mature green (MG), and ripe (R)). Values are normalized to mean value of wild type at 22 DAP stage. Data showed as means ± SE of 5 individual plants. Asterisks indicate statistically significant differences determined by Student *t* test (*P* < 0.05) of the transgenic line compared with wild type at the same developmental stage.

**Figure 5:**
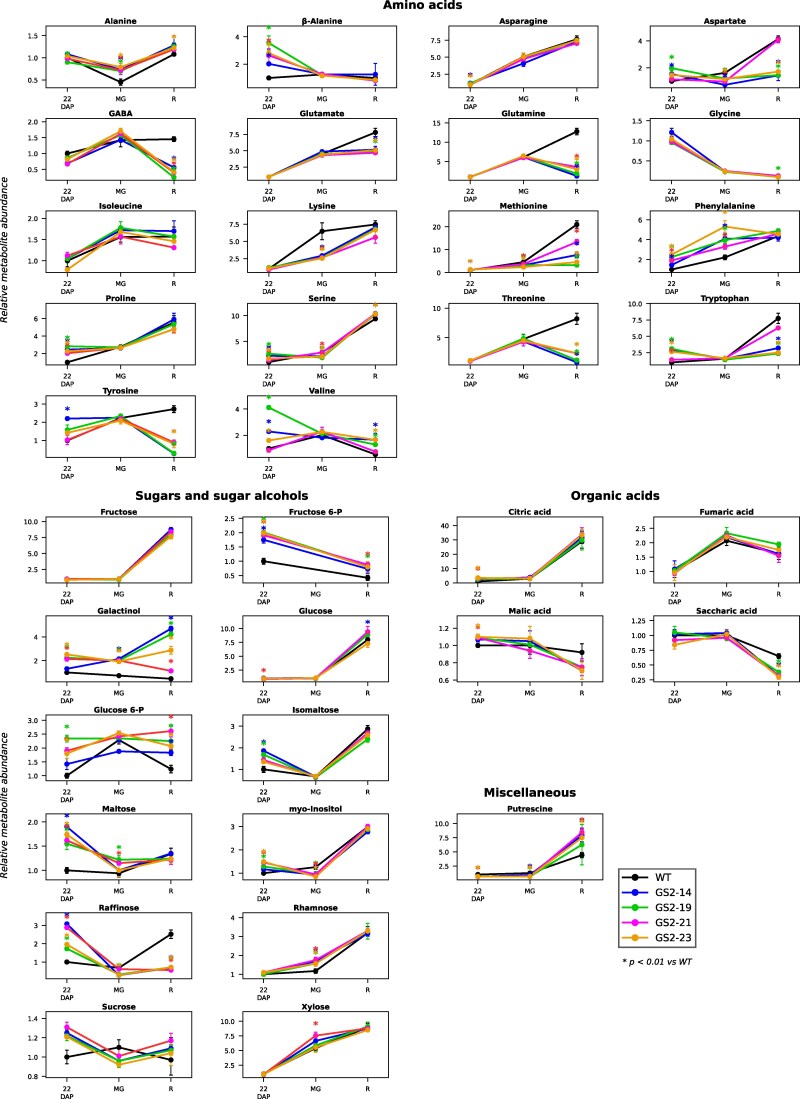
Primary metabolites measured in tomato fruits of wild-type and RbcS–GS2 lines at 3 developmental and ripening stages (22 d after pollination (DAP), mature green (MG), and ripe (R)). Values are normalized to mean value of wild type at 22 DAP stage. Data showed as means ± SE of 5 individual plants. Asterisks indicate statistically significant differences determined by Student *t* test (*P* < 0.05) of the transgenic line compared with wild type at the same developmental stage.

The changes in primary metabolism during fruit development and ripening in the transformants were analyzed by using the same protocol as for leaves. We evaluated the metabolite levels in fruits harvested 22 d after pollination (DAP) and at mature green (MG) and ripe (R) stages. In general, similar patterns of changes were observed when comparing the RbcS-COX4ts-GS1 and RbcS-COX4ts-GS2 lines. At the ripe stage, both lines exhibited an increase in most of the free amino acids, including serine, threonine, alanine, ß-alanine, proline, valine, glycine, and aspartate compared with control fruits (Figs. 4 and 5). However, the levels of glutamine, glutamate, tyrosine, GABA, and methionine was significantly lower in the transgenic lines at the ripe stage. We additionally observed significant increases in the levels of sucrose at 22 DAP and in ripe fruit (between 1.2- and 1.4-fold of wild-type levels at 22 DAP and between 1.8- and 2.2-fold of wild-type levels at the ripe stage), although no significant changes were observed in the GS1-2 line. Similarly, an increase in the levels of sugar phosphates, glucose-6P and fructose-6P, was observed in all lines. Interestingly, these changes were coupled with an increase in the level of starch at 22 DAP stage in all transgenic lines (235.2 ± 18.5; 255.2 ± 15.3; 387 ± 20.4; 315.2 ± 11.8; 375.4 ± 15.8; 336.7 ± 21.4; 367.2 ± 17.3; 299.4 ± 12.1; 287.4 ± 16.4 nmol g^−1^ DW for control, GS1-2, GS1-3, GS1-6, GS1-33, and GS2-14, GS2-19, GS2-21, GS2-23, respectively). Two disaccharides associated with starch degradation, maltose and isomaltose, displayed slightly increased levels at the 22 DAP stage in all lines, while isomaltose levels were lower at mature green and ripe stages in the transgenics compared with the control (Figs. 4 and 5). These changes were accompanied by increases in the other major sugars, namely fructose and glucose, that also increased at ripe stage. By contrast, the levels of saccharate and raffinose, products released following glucose oxidation, were lower at the ripe stage in the transgenic lines (Figs. 4 and 5). Perhaps surprisingly given that significant differences were found in the levels of major sugars in ripe fruits, we did not observe significant differences in the total soluble solids content. Despite the levels of fumarate and malate being invariant in the fruit of the transgenics, other metabolic changes of note were that citrate was observed at significantly higher levels at the ripe stage in all lines. Furthermore, the levels of the cell wall–related sugars, xylose and rhamnose, which increase during fruit ripening consistent with the known increased cell wall degradation at this time point, were higher in the transgenic lines in comparison to control fruits (Figs. 4 and 5). Moreover, significantly lower putrescine levels were observed at both 22 DAP and mature green stages, but significantly higher levels were observed in ripe fruit in all transgenic lines (Figs. 4 and 5).

### Effect of increasing mitochondrial GS activity in leaves on metabolic fluxes in the fruits

**Figure 6:**
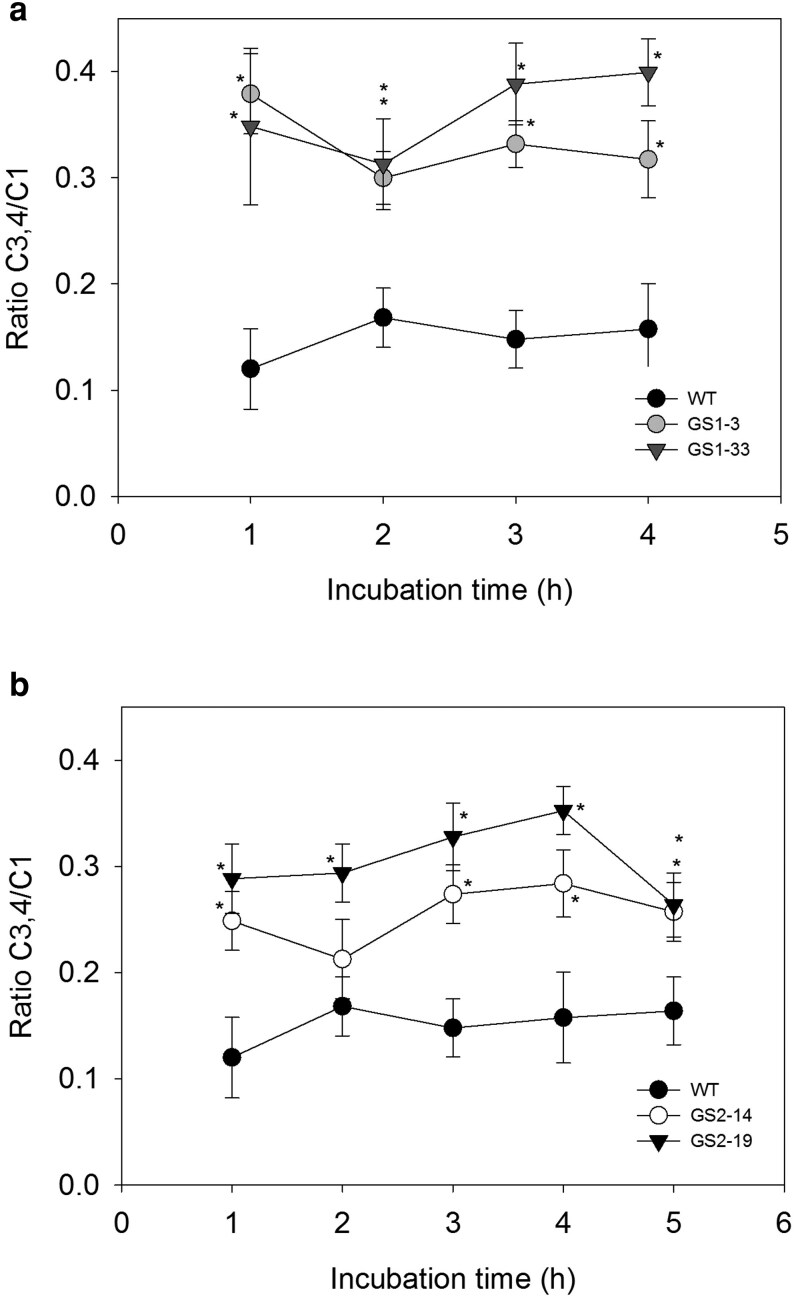
Ratio of CO_2_ evolution from C3.4/C1 positions of glucose in pericarp fruits of 22 DAP stage from (a) *RbcS–GS1* and (b) *RbcS–GS2* lines.

Given the changes in sugars and starch levels in small fruits (22 DAP), we next decided to assess the relative rate of carbon fluxes in the lines with the highest expression levels by incubation of fruit pericarp discs at 22 DAP stage of control and 2 GS1 lines (GS1-3 and GS1-33) and 2 GS2 lines (GS2-14 and GS2-19) with positionally labeled ^14^C-glucose. Samples were incubated in [1-^14^C]-glucose and [3,4-^14^C]-glucose for 5 h, and the ^14^CO_2_ evolution was monitored. The CO_2_ release from C1 of glucose is associated with respiration through the oxidative pentose phosphate pathways, while the respiration through TCA cycle is linked to release from C3,4 position of glucose. Therefore, a high ratio of CO_2_ evolution from C3,4 to the C1 position of glucose provides strong evidence of mitochondria activity. When comparing the ^14^CO_2_ releases from control and RbcS-COX4ts-GS1 and RbcS-COX4ts-GS2 lines, we observed a significant increase in the C3:4/C1 ratio in transgenic fruits (Fig. 6).

To expand this study, same samples were incubated with [U-^14^C]-glucose for 3 h to assess the redistribution of radiolabel. We did not observe changes in total label incorporated; however, differences were apparent when comparing radiolabel redistribution (Table 4). In agreement with the steady-state metabolite data, significantly increased label incorporation into starch and the total hexose phosphate pool was observed in the analyzed lines at the 22 DAP stage (lines GS1-3, GS1-33, GS2-14, and GS2-19). However, no significant changes were observed in either label redistribution to cell wall, organic acids, amino acids or sucrose (Table 4). This pattern was also seen following estimation of the fluxes with an elevated rate of starch synthesis being the only consistent change, although it is important to note that the rate of amino acid biosynthesis was also observed to slightly decrease in line GS1-3.

**Table 4.** Changes in respiratory carbon redistribution of radiolabel carbon after incubation of ^14^C-Glc in pericarp discs from RbcS-COX4ts-GS1 and RbcS-COX4ts-GS2 lines at 22 DAP stage.

Parameter	Wild-type	GS1-3	GS1-33	GS2-14	GS2-19
Label incorporated (Bq)					
Total uptake	380.8 ± 34.5	382.3 ± 23.8	397.5 ± 47.3	379.3 ± 38.2	401.4 ± 38.6
Cell wall	8.5 ± 0.6	8.1 ± 0.9	7.7 ± 0.7	9.1 ± 1.0	8.6 ± 0.8
Starch	55.6 ± 7.7	**72.6** ± **9.5**	**78.2** ± **7.5**	**66.6** ± **4.6**	**70.5** ± **6.6**
Organic acids	150.6 ± 11.7	132.5 ± 8.7	137.4 ± 9.3	137.3 ± 10.3	150.5 ± 9.3
Total hexose phosphate	40.7 ± 9.4	**55.8** ± **5.3**	**52.4** ± **6.3**	**64.5** ± **8.3**	46.3 ± 9.3
Amino acids	88.6 ± 7.3	79.6 ± 9.5	93.5 ± 8.7	85.7 ± 6.9	90.6 ± 11.5
Suc	33.6 ± 4.8	30.6 ± 6.7	25.5 ± 5.7	23.7 ± 7.9	32.7 ± 6.9
Metabolic flux (nmol g^−1^ DW h^−1^)					
Cell wall	85.6 ± 7.5	77.6 ± 8.3	91.7 ± 9.2	80.7 ± 7.7	95.8 ± 8.4
Starch	167.8 ± 11.7	**247.4** ± **20.6**	**210.4** ± **15.3**	**196.7** ± **9.4**	**216.5** ± **11.5**
Organic acids	306.7 ± 18.7	275.5 ± 23.7	269.4 ± 22.7	315.6 ± 16.3	289.4 ± 24.3
Amino acids	278.3 ± 12.4	**238.4** ± **9.5**	262.6 ± 13.5	296.6 ± 13.5	275.5 ± 7.8
Suc	57.3 ± 8.4	68.3 ± 12.5	50.7 ± 9.7	73.7 ± 7.7	63.4 ± 8.3

Values set in bold text are significantly different from the wild type as determined by Students t-tests (*P* < 0.05).

To integrate the phenotypic measurements obtained across the transgenic lines, we performed a line-level correlation analysis of the traits recorded throughout this study (Fig. S9). Transgene expression and total fruit weight per plant were strongly and positively correlated (ρ = 0.97, *P* < 0.001), and both variables also correlated positively with total GS activity (ρ = 0.82 and ρ = 0.73, respectively; *P* < 0.01 and *P* < 0.05). Both temporal traits, weeks from transplanting to first anthesis and days from anthesis to first ripe fruit, were negatively associated with yield (ρ = −0.89 and ρ = −0.90; *P* < 0.01) and with GS activity (ρ = −0.83 and ρ = −0.77; *P* < 0.01 and *P* < 0.05), indicating that earlier reproductive transitions accompanied higher GS activity and higher yield. Notably, stem and root dry weights did not correlate with yield (*P* > 0.7), arguing that the yield gain in the transgenic lines does not derive from increased structural biomass. Given the limited number of lines (n = 9), these analyses should be interpreted as exploratory and corroborative of the trait-by-trait comparisons reported above.

## Discussion

During recent decades, considerable effort has been dedicated to elucidating molecular mechanisms associated with enhanced biomass. N and C metabolism are tightly coordinated in the fundamental biochemical pathways that support plant growth (Nunes-Nesi et al. 2010; Perchlik and Tegeder 2018; Lu et al. 2020). Understanding of the regulation of these pathways is therefore essential for optimizing plant growth can improve crop production (Sonnewald and Fernie 2018; Fernie et al. 2020; Sonnewald et al. 2020). As such, many different studies have focused on N metabolism and C/N interactions (Perchlik and Tegeder 2018; Lu et al. 2020; Vallarino et al. 2020), with a couple of these attempting to use multi-gene approaches in order to influence both C and N metabolism simultaneously (Zhang et al. 2015; Vallarino et al. 2020). Here, we postulate, as previously described, that plant metabolic pathways change to be adaptable to combat different environment conditions (Bohnert et al. 1995), although sometimes this adaptation results in yield losses (Amthor et al. 2019). In this study, we used constraint scanning of an FBA leaf model to predict metabolic flux distributions associated with an increase in metabolic efficiency in leaves. The simulations showed a potential mechanism to improve leaf metabolic efficiency and increase biomass with reduced energy costs was to move a functional glutamine synthetase (GS) into the mitochondria. GS catalyzes the ATP-dependent biosynthesis of Gln from Glu and NH_4_^+^. There are 2 GS isoforms, a cytoplasmic form (GS1) and a plastidic form (GS2). GS2 is described as involved in the re-assimilation of NH_4_^+^ produced during photorespiration and nitrate reduction while GS1 has been associated to the assimilation of NH_4_^+^ produced from other physiological processes (Seger et al. 2015). Consistent with a physiological role for mitochondrial GS activity, previous work in Arabidopsis has shown that GS2 can be dual-targeted to plastids and mitochondria, with mitochondrial GS activity induced in response to CO_2_ limitation or transient darkness (Taira et al. 2004; Hooper et al. 2017). While the native subcellular distribution of SlGS2 in tomato remains to be fully characterized, our confocal microscopy data confirm that the COX4ts-fused transgene products are exclusively localized to the mitochondria (Fig. S2a–d).

Here, we evaluated the effect of independently overexpressing the 2 GS isoforms in mitochondria under the control of the leaf mesophyll-specific promoter of ribulose-bisphosphate carboxylase (RbcS). This allowed us to assess whether mitochondrial manipulation of GS activity produced the physiological changes in tomato plants that we anticipate from the model. The results obtained indicated that modified mitochondrial GS activity can indeed result in altered plant development due to elevated source to sink transport. The observed changes included increased and earlier flowering and fruit set which culminated in both a shortened production cycle and an enhanced yield.

### Effects of overexpression of a mitochondrially targeted GS on leaf metabolism

A considerable amount of research has long suggested that N assimilation and remobilization are important interconnected processes that play key roles in plant growth and development (Miflin and Habash 2002). Here, we provide experimental evidence that the mitochondrially targeted overexpression of *GS1* or *GS2* in mitochondria led to similar alterations in plant growth and development. When taken together, our data suggest that shifting GS activity to mitochondria, renders leaves more efficient with regard to their N assimilation/mobilization pathways.

Irrespective of the gene used the increased GS activity in mitochondria of the transgenics resulted in an enhanced rate of early plant growth with plants also producing more fruits. This phenomenon demonstrates that shifting the GS activity to the mitochondria can improve the efficiency of leaf metabolism, thereby improving the source strength of the leaves. This observation was in concordance with higher values of shoot and root biomass together with a notable rise in the content of major amino acids and total soluble proteins in leaves (which represent the major source of organic nitrogen in plants).

The use of 2 biochemically distinct GS isoforms strengthen the generality of our findings. SlGS1 (Solyc04g014510) and SlGS2 (Solyc01g080280) share 78.65% protein sequence identity but differ in native subcellular localization (cytosolic vs plastidic), subunit molecular mass (∼39 vs ∼44 kDa), and physiological function: GS1 is primarily involved in primary nitrogen assimilation and nitrogen remobilization, while GS2 is mainly associated with reassimilation of photorespiratory ammonium (Bernard and Habash 2009). The convergence of equivalent phenotypes from 2 biochemically distinct proteins when targeted to mitochondria indicates that the observed effects are attributable to increased GS catalytic capacity within this compartment rather than to isoform-specific biochemical properties.

The specificity of the mitochondrial compartment for the observed phenotypes was further confirmed by the generation of control lines expressing GS1 in the cytosol and GS2 in the plastid without mitochondrial targeting. Despite substantial increases in total GS activity (up to 60-fold in cytGS1 lines), these control plants showed no alterations in growth, flowering, or fruit yield compared with wild type (Figs. S3 and S4). This result is particularly striking in the case of the cytGS1 lines, where a 60-fold increase in total cellular GS activity produced no detectable phenotype, while even modest increases in mitochondrial GS activity (1.2- to 1.8-fold) were sufficient to enhance plant growth and fruit production. These findings reinforce the conclusion that the metabolic advantage conferred by GS activity is strictly dependent on its subcellular localization within the mitochondria, in agreement with the predictions of the diel FBA model.

These observations sit within a broader landscape of attempts to manipulate GS for improved nitrogen use efficiency and yield in crops, which has produced heterogeneous outcomes (Thomsen et al. 2014; Liu et al. 2022). While some strategies overexpressing native or heterologous GS isoforms in their canonical compartments have delivered yield gains in cereals, including plastidic GS2 in wheat (Hu et al. 2018) and cytosolic GS1 in rice (Wu et al. 2021), others have failed to translate increased GS abundance or activity into productivity benefits, in part because GS is tightly modulated by post-translational mechanisms responsive to metabolic and environmental status (Thomsen et al. 2014); a recent example in tomato is the phosphorylation of plastidic GS2 by the phytosulfokine receptor signaling pathway, which uncouples growth from defense responses (Ding et al. 2023). Whether such post-translational regulatory layers intersect with mitochondrially targeted GS remains to be established. The present results extend this body of work in a distinctive direction: rather than amplifying GS activity in its native compartment, we redirect it to the mitochondrial matrix, where it intersects respiratory carbon flux and the cytosolic provision of ATP. The relevance of mitochondrial metabolism for whole-plant nitrogen homeostasis and source-sink balance has been recently underscored by studies linking respiratory activity to amino acid biosynthesis and growth (Vera-Vives et al. 2025), providing an independent line of evidence consistent with the mechanism proposed here.

Interestingly, despite displaying apparently elevated source strength, the transgenic lines did not display major changes in photosynthetic parameters—yet were characterized by lower levels of soluble sugars and starch than the wild-type control. This finding indicates that the transgenics are likely characterized by a higher C export to sink organs. This raises the likelihood of an increased flux of trioses-Ps from the chloroplast to the cytosol, which via previously defined mechanisms (Heldt and Flugge 1987; Stitt 1990a) would increase the cytosolic hexoses-P pool and thereby drive a higher rate of Suc synthesis. Consistent with this hypothesis is the fact that we observed higher Suc levels in leaf exudates as well as reduced enzymatic activities of phosphoglucomutase (PGM) and ADP-glucose pyrophosphorylase (AGPase) in the leaves of both sets of transgenic plants. Given that these enzymes have been documented to play key roles in starch biosynthesis (Sweetlove et al. 1999; Fernie et al. 2001), which is essentially competitive to sucrose biosynthesis in the illuminated leaf (Stitt 1990b), these data fully fit our proposed FBA model results. Furthermore, AGPase activity appears also to be modulated at the post-translational level (Hendriks et al. 2003). One of the regulatory mechanisms coordinating the activity of this enzyme is associated to the plastidial redox state, with malate playing a key role in mediating intra-organellar redox metabolism (Centeno et al. 2011; Szecowka et al. 2012) Interestingly, overexpression of GS1 and GS2 in mitochondrial produced a significant increase in the NADPH level altering the NADPH/NADP^+^ level in photosynthetically active leaves, which is also consistent with altered AGPase activity in these lines. These transgenic plants also showed altered malate levels in leaves. Therefore, the results are strongly in agreement with previous evidence that starch metabolism is modified by redox active intermediates levels including malate (Centeno et al. 2011; Osorio et al. 2013).

In addition to the above-described effect of mitochondrial GS1 or GS2-overexpression, 2 important observations can be highlighted in the transgenic leaves in comparison with wild type: 1) an activation in the initial and total activities of the NADP-dependent malate dehydrogenase (NADP-MDH) of the chloroplast, which uses excess NADPH to convert oxaloacetate to malate; and 2) a slight increase in photosynthetic pigment accumulation. Both observations have been commonly used as diagnostic markers for alterations in plastidial redox status (Scheibe 1991; Scheibe et al. 2005; Nashilevitz et al. 2010). However, we cannot exclude that these changes in chlorophyll content can be due to the observed alteration in the level of the amino acid Glu in the leaves of the transformants, since Glu is the precursor for tetrapyrrole ring biosynthesis (Von Wettstein et al. 1995; Lytovchenko et al. 2011).

Our results suggest that the metabolic signaling-network mechanisms that allow C and N to be remobilized from leaves to increase biomass and fruit production by modifying mitochondrial GS activity may be associated with changes in the redox state in leaves. In this vein, Chaouch et al. (2010) previously suggested that photorespiration plays a key role in the regulation of cellular redox status. Photorespiration, furthermore, interacts with a range of diverse metabolic processes including but not limited to photosynthesis, N metabolism, respiration (Foyer et al. 2009; Bauwe et al. 2010; Bloom et al. 2010; Fernie et al. 2013) and has previously been positively associated with productivity (Aliyev 2012). In this study, we observed indicators of an increased photorespiratory flux in that we detected a significant increase mRNA levels of the L subunit of the glycine decarboxylase complex (GDC) and an accumulation in Ser levels. The higher rate of photorespiration in the transgenic plants together with the increased NADP-MDH activity described above is suggestive of activation of the malate valve to transport the excess of reducing equivalents out of chloroplast. In keeping with this conclusion are the previous results demonstrating that tobacco leaves displaying reduced expression of NADP-MDH were also characterized as displaying decreased rates of photorespiration (Backhausen and Scheibe 1999).

Previous studies have also described a close relation between photorespiration and 2-oxoglutarate (2-OG) metabolism (including both the TCA cycle and GABA shunt) (Eisenhut et al. 2013; Carroll et al. 2015; Fromm et al. 2016). Our results here suggest that functions of chloroplasts and mitochondrial compartments are tightly coordinated through redox and intracellular metabolite pool exchanges as previously described (Nunes-Nesi et al. 2005; Noguchi and Yoshida 2008; Araújo et al. 2012; Osorio et al. 2013). Our results also demonstrated a marked shift in 2-OG metabolism. Evaluation of the steady-stage metabolite levels were, by and large, in keeping with a reduction in flux through the mitochondrial steps of the TCA cycle in that the TCA intermediates succinate, fumarate, and malate were reduced in the leaves with increased mitochondrial GS activity. By contrast, the levels of 2-OG and citrate were dramatically increased. Interestingly, we also noticed altered levels of several amino acids derived from 2-OG such as Glu, Pro, and Gln. Although our results suggested a substantial suppression of TCA cycle activity, this was not reflected in the maximal catalytic activities of its constituent enzymes. However, this is not overly surprising since previous studies have suggested that these enzymes are present in levels vastly in excess of what is needed in terms of the fluxes they bear (Gibon et al. 2004).

The observed changes in different accumulation in metabolites directly related to N assimilation pathway, especially Glu, Pro, Gln, Asn, Arg, and 2-OG levels prompt the question: how can the TCA cycle provide enough carbon skeletons to increase N assimilation rather than synthesis of ATP? With the observation of higher citrate levels in all transgenic lines (Fig. 3), it could be postulated that this citrate, which includes citrate accumulated in leaf vacuoles during the night, could contribute to the increased C skeleton demand for N assimilation. At the same time, the availability of NADH from glycine decarboxylase flux (photorespiration) should be sufficient to support the mitochondrial electron transport flux required for ATP synthesis. In this study, the activity of N-assimilatory enzymes in transgenic leaves displayed interesting results. As expected, we detected higher GS activity, but also we observed a significant increase in nitrate reductase (NR) and glutamate dehydrogenase (GluDH) while nitrite reductase (NiR) and glutamate synthase (GOGAT) were decreased in the transgenic plants. Our results thus underline the previously described (Hodges 2002), strong links between 2-OG metabolism and N assimilation.

### Effects of mitochondrially targeted overexpression of GS on flowering, fruit set, and fruit metabolism and yield

As a sink tissue, fruit strength is largely dependent on C supply from the phloem, especially in the form of Suc (Zrenner et al. 1995; Sonnewald et al. 1997; Fridman et al. 2004; Obiadalla-Ali et al. 2004). Our data indicate that mitochondrial GS activity improves N assimilation and increases photoassimilate partitioning and transport to fruit. This had several agronomically important consequences, namely the transgenic tomato plants flowered earlier and more frequently, and fruit set was correspondingly also earlier and more frequent. Given that at least at northern latitudes most tomatoes are grown year-round in greenhouses, this in itself could constitute a major gain since it may allow an increased number of harvests per year. The results presented here suggest that despite displaying earliness in growth rate and flowering, the fruit size was similar or even smaller that the wild type. That said, the mitochondrially targeted overexpression of GS result in enhanced overall tomato yield, which suggests that the model predicted a highly useful intervention.

We also observed a higher rate of starch accumulation rate during the fruit growth stage in the transgenic plants that, as we have seen previously (Centeno et al. 2011), corresponded to increased Glc, Fru, and Suc levels in ripe fruits. Consistent with these changes in the starch accumulation at the 22 DAP stage from both lines was the increase in the starch biosynthetic flux and also increased label into pool of hexose phosphate. These data also support previous observations that starch accumulation during fruit development is an important source of sugars in ripe fruits (Baxter et al. 2005; Osorio et al. 2013; Colombié et al. 2015). It is important to remember here that the genetic intervention was leaf mesophyll specific, so the fact that altered flux was maintained in isolated pericarp discs from the transformants suggests that the latency of these differences is caused by a reprogramming of sink metabolism as a consequence to changes in the source. This observation leads us to propose that an elevated sink strength at least partially drove the higher sugar influx from source leaves into transgenic fruits, increasing the rate of starch synthesis and consequently the soluble sugar levels during fruit ripening. It will be interesting in future studies to address the precise mechanism by which this is achieved.

We additionally observed higher levels of the cell wall–associated sugars xylose and rhamnose during fruit ripening in both transformants, which may indicate higher degradation rate of the cell wall pectin; however, the results from the feeding experiments did not indicate changes in the total label incorporated to the cell wall. Other metabolic changes found in ripe fruit of the transformants, such as increase of amino acids, potentially reflect a higher protein degradation rate at late ripening stages in order to support the increased respiration rate through the TCA found in ripe fruit in both sets of transformants. In addition, a significant accumulation of putrescine was observed in the ripe fruits from both lines. Putrescine is one of the most prevalent polyamines in plants and has been implicated in alkaloid biosynthesis as well as being a mediator of several signaling pathways (Bienz et al. 2005; Mattoo and Handa 2008). Catabolism of polyamines has been described to feed TCA cycle via the GABA pool (Rastogi and Davies 1991; Bienz et al. 2005). However, further research is needed to ascertain the significance of these changes.

In conclusion, we demonstrate here that overexpressing a targeted GS activity in leaves is an effective strategy for improving leaf metabolic efficiency and both the number of fruit set, the earliness of this developmental transition, and ultimately an enhanced fruit yield. In light of growing interest in urban farming (Fernie and Yan 2020; Kwon et al. 2020) alongside the fact that a large proportion of global tomato growth occurs in greenhouses, the earliness phenotype alone is likely of high agronomic value as the shortening of the production cycle potentially allows a greater number of harvests per year. Our results provide evidence that by moving the GS activity into mitochondria in leaves, we observed a strong effect on the source strength of the leaves that were more efficient in N assimilation/mobilization. It appears that the higher fruit production observed was due to altered photoassimilate distribution. Indeed, despite unaltered photosynthetic rates, there was a marked shift in carbon partitioning from starch to sugars in the transformants. However, sugars did not greatly accumulate since this was offset by an even greater increase in the loading of assimilates into the phloem. This is an ideal scenario since it circumvents the well characterized phenomenon of feedback inhibition of photosynthesis whereby high leaf sucrose levels suppress the rate of photosynthesis (Stitt 1990a). In fruits, we demonstrated that the higher sugar influx from leaves led to higher sugar levels in ripe fruit, although significant differences in the main sugars did not correspond to an enhanced Brix index, most likely due to corresponding decreases in other components of this composite trait. While enhancing mGS activity into leaf metabolism did not improve CO_2_ assimilation rate, we demonstrated how metabolic modeling studies can lead to hypotheses generating non-obvious targets for increased crop yield, suggesting that the recently established crop in silico project (https://cropsinsilico.org/) will likely provide considerable novel hypotheses that will ultimately aid us in the metabolic engineering of plants. In our case it suggested that the enhanced mitochondrial activity of glutamine synthetase would represent a route to optimize phloem loading. Our experimental data from plant engineered with this suggested intervention revealed that this was indeed the case and that as a consequence tomato plant expressing either tomato isoform of this enzyme displayed early and increased incidence of flowering, alongside an early fruit set and a shorter production cycle. The fact that at more northern latitudes most commercial tomato production occurs in greenhouses, moreover, renders the early setting of fruit of potentially highly useful trait. Even more so given that the final fruit yield is additionally enhanced. As such, this finding was perhaps surprisingly far more successful than our previous attempts to achieve this using multi-gene approaches (Vallarino et al. 2020).

## Materials and methods

### Flux balance analysis modeling tomato leaf metabolism

The model PlantCoreMetabolism v2.0.0 was based on updated version of our previously described core model of plant metabolism (Shameer et al. 2020) and modified to include gene-to-reaction associations for tomato. A curation log specifying the updates to the model is provided in Data Set S4. Gene association data were gathered from PlantCyc Tomato Pathway/Genome Database (www.plantcyc.org). Data from the cropPAL database (Hooper et al. 2016) were used to ensure gene-associations were in line with known enzyme subcellular localization. The model is provided as an SBML file (Data Set S5). A 2-phase day-night (diel) formulation was established as previously described (Cheung et al. 2014). The CO_2_ assimilation rate of the model was constrained using the approach previously described (Shameer et al. 2019). The model output was a defined stoichiometry of sugars and amino acids representing tomato phloem sap composition (Walker and Ho, 1977; Valle et al. 1998). Non-growth–associated maintenance was set according to the incident light flux following a previously described procedure (Töpfer et al. 2020). Other model constraints are listed in Table S1. The model was run using the COBRAPy package (Ebrahim et al. 2013) for Python 3. The built-in COBRAPy functions for pFBA and FVA were used to solve the model. A constraint scan on the model output was done by reducing the model output in 2% steps and each time re-running the model with this constrained output and storing the resulting flux solution. Fluxes were graphically plotted using the python package Matplotlib. All code required to run the constraint-scan and generate plots can be found in https://github.com/sshameer/Vallarino_et_al_2021.

### Plasmid construction and generation of transgenic plants

Transformation of tomato plants (*Solanum lycopersicum* cv MoneyMaker) was performed essentially as described in (Nunes-Nesi et al. 2005). Two pK2GW7 plasmids were prepared containing the *nptII* gene for kanamycin resistance, the mitochondrial transit peptide COX4ts (Nelson et al. 2007), and the full-length *S. lycopersicum Glutamine Synthetase* (*GS*) 1 and 2 genes [*GS1* (*Solyc04g014510*) and *GS2* (*Solyc01g080280*)] in sense orientation under the control of the leaf mesophyll-specific promoter (RbcS). We screened 10 and 14 independent transgenic tomato lines harboring *RbcS-COX4ts-GS1* and *RbcS-COX4ts-GS2* constructs, respectively, based on their levels of *GS1* and *GS2* gene expression. We subsequently selected 4 lines per construct that were taken to the next generation (T_1_) for detailed characterization. Transgenic plants were grown in greenhouse conditions under a 16-/8-h photoperiod, 60% RH, and light intensity of 400 µmol m^−2^ s^−1^ as previously described (Nunes-Nesi et al. 2005).

### Generation of cytosolic GS1 and plastidial GS2 control lines

To generate control lines without mitochondrial targeting, the same full-length cDNAs encoding GS1 and GS2 were cloned in the sense orientation into the pK2GW7 vector under the RbcS promoter but without the COX4ts mitochondrial transit peptide. This directs GS1 to its native cytosolic compartment (cytGS1 lines) and GS2 to the plastid (plGS2 lines). We screened 12 and 16 independent transgenic tomato lines for cytGS1 and plGS2, respectively, based on PCR confirmation of transgene insertion. Five independent lines per construct were selected for further characterization.

### RNA extraction and gene expression by RT-qPCR

Total RNA was isolated from leaves according to the method described by (Sánchez-Sevilla et al. 2017) Gene expression in tomato leaves was analyzed by real-time quantitative RT-PCR using the fluorescent intercalating dye SYBR Green. Relative quantification of the target genes was performed using the comparative Ct method. Expression data were normalized to the reference genes Ubiquitin3 and elongation factor with comparable results. Values displayed in this publication are normalized to Ubiquitin3 (Wang et al. 2008; Zanor et al. 2009). Primers used are detailed in Table S2.

### Photosynthetic parameters

Leaf gas exchange and chlorophyll fluorescence were performed using a Li-6400XT (Li-Cor Inc., Lincoln, NE, USA). All measurement conditions were as described by (Vallarino et al. 2020).

### Metabolite analysis

The level of starch in 100 mg of leaves and 250 mg of fruits, respectively, were extracted and analyzed spectrophotometrically as described previously (Dominguez et al. 2013). Starch was determined in the third fully expanded leaf from the apex, harvested at midday. Pyridine nucleotide levels were performed according to (Schippers et al. 2008). Photosynthetic pigments were determined according to the methods described by Porra and Scheer (2009). Primary metabolites were carried out in leaves, phloem exudates and pericarp of fruits at different development and ripening stages by using gas chromatography coupled to electron impact ionization-time of flight-mass spectrometry (GC-EI-TOF/MS) according to method described in (Lisec et al. 2006). Absolute metabolite levels in phloem exudate were measured in samples obtained as described in (Lytovchenko et al. 2002), by running standard curves of authentic standards for quantification. Metabolite levels are reported following the reporting standards suggested by (Fernie et al. 2011; Alseekh et al. 2021; Data Set S6).

### Mitochondrial isolation

Mitochondria were isolated from 2-mo-old tomato leaves as described by Kerbler and Taylor (2017). Briefly, ∼30 g of whole seedlings was disrupted in a precooled mortar and pestle using 150 mL of cold extraction buffer (0.3 M sucrose, 25 mM tetrasodiumpyrophosphate [10 H2O], 2 mM EDTA, 10 mM KH2PO4, 1% polyvinylpyrrolidone [PVP-40], 1% BSA, 20 mM ascorbic acid, 5 mM cysteine [pH 7.5]). The homogenate was filtered twice through 4 layers of Miracloth (CalBioChem). The material that was retained was recovered and re-grounded with 100 mL extraction buffer. This step was repeated 3 times. The filtrate was first centrifuged at 2,500 *g* for 5 min to separate the chloroplast (pellet) and mitochondrial (supernatant) fractions. The supernatant containing the mitochondria-enriched fraction was further centrifuged for 15 min at 18,000 *g*. The pellet was resuspended in a small volume of washing buffer (0.3 M sucrose, 10 mM TES-KOH, and 0.1% [w/v] BSA [pH 7.5]). An additional 35 mL of washing buffer was added, and the mixture was centrifuged again at 2,500 *g* for 5 min. The supernatant was transferred to new tubes (40 mL), making sure that no pellet was also carried over. The suspension was then centrifuged at 18,000 *g* for 15 min. The obtained pellet was resuspended in 2 mL of washing buffer and loaded on top of a 28% (v/v) Percoll (GE Healthcare) continuous gradient with a 0% to 4% (w/v) PVP-40 linear gradient and centrifuged at 38,000 *g* for 50 min. The mitochondrial band was collected, washed 3 times in sucrose wash buffer (without BSA) (0.3 M sucrose, 10 mM TES-KOH [pH 7.5]) and divided into aliquots for measuring enzyme activity.

Purified mitochondria were lysed in the extraction buffer (extraction buffer: 8.7% [v/v] glycerol, 0.5% [w/v] BSA, 1% [v/v] Triton X-100, 50 mM 4-(2-hydroxyethyl)-1-piperazineethanesulfonic acid (HEPES)/KOH pH 7.5, 10 mM MgCl2, 1 mM ethylenediamine tetraacetic acid (EDTA), 1 mM ethylene glycol tetraacetic acid (EGTA), 1 mM benzamidine, 1 mM ɛ-aminocaproic acid, 10 mM phenylmethylsulfonyl fluoride (PMSF) in isopropanol, 0.2 mM leupeptin, and 50 mM dithiothreitol) for mitochondrial enzyme activity measurement (Omena-Garcia et al. 2017).

### Enzyme activities

#### GS

Enzyme activity was measured by the synthetase reaction according to Machado et al. (2001). GS activity in the cytGS1 and plGS2 control lines was measured according to Gibon et al. (2004) and Ménard et al. (2014).

#### GOGAT

The GOGAT assay was adapted from (Groat and Vance 1981) and measured as rate of glutamine-dependent NADH oxidation at 340 nm for 3 min. Extracts were incubated in a solution containing 25 mM KH_2_PO_4_ buffer (pH 7.5), 1 mM hydroxylamine, 2.5 mM 2-oxoglutarate, 0.1 mM NADH, and approximately 100 μg of total protein. The reaction was started by the addition of 10 mM of glutamine.

#### GDH

GDH was measured as described by (Robinson et al. 1991), as the rate of 2-oxoglutarate-dependent NADH oxidation at 340 nm for 3 min.

#### PGM, PEPC, NAD-MDH, and NADP-MDH

Enzyme extracts were prepared as described previously by (Gibon et al. 2004).

#### AGPase

Enzyme activity was assayed as described in (Osorio et al. 2013).

#### NR and NiR

NR was determined as decreased absorbance at 340 nm due to NADH oxidation as described in (Campbell and Smarrelli 1978). NiR activity was measured exactly as described previously (Lillo and Ruoff 1992). The absorbance was read at 540 nm at 25 °C.

#### SDH and UGPase

To assess organelle purity, we measured succinate dehydrogenase (SDH) activity as a mitochondrial marker and UDP-glucose pyrophosphorylase (UGPase) activity as a marker of cytosolic contamination (Omena-Garcia et al. 2017).

### Analysis of the redistribution of positionally labeled Glc

TCA cycle flux analysis was calculated based on ^14^CO_2_ evolution. Fruit pericarp disks at 22 DAP stage from transgenic lines and wild type (10 disks per biological replicate. A total of 5 replicates were used) were incubated in 5 mL of 10 mM MES-KOH, pH 6.5 containing 2.85 kBq ml^−1^ of [1-^14^C]- or [3,4-^14^C] Glc. Evolved ^14^CO_2_ was collected in 0.5 mL KOH (10% w/v) every hour (for 5 h) and quantified by liquid scintillation counting. Disks were washed 5 times in 10 mM MES-KOH, pH 6.5 and frozen in N_2_ liquid for further analysis. The ^14^C-labeled material was fractionated according to (Lytovchenko et al. 2002). The results were interpreted following recommendations detailed by (Geigenberger et al. 2000).

### Agrobacterium culture preparation and infiltration for mitochondrial localization

The agrobacteria were grown on YEB-induced medium plates (Zhang et al. 2020) (at 28 °C for 24-36 h. The cells were scratched and resuspended in 500 µL washing solution (10 mM MgCl_2_, 100 μM acetosyringone). After briefly vortexing, the 100 µL resuspended agrobacteria were diluted 10 times to measure the OD600 (should be equal to or greater than 12). The agrobacteria were finally diluted to OD600 = 0.5 in infiltration solution (¼MS [pH = 6.0], 1% sucrose, 100 μM acetosyringone, 0.005% (v/v, 50 μL/L) Silwet L-77). The agrobacteria were infiltrated into tobacco leaves kept in the light to dry the leaves (1 h), using a 1 mL plastic syringe, and then subsequently kept in the dark for 24 h at room temperature. The transformed plants were then transferred back to the greenhouse for another 2 to 3 d before microscopy. Confocal images were taken using a DM6000B/SP8 confocal laser scanning microscope. GFP and mCherry fluorescence were imaged with 495- and 565-nm laser excitation, and emission fluorescence was captured by 505- to 520-nm and 600- to 630-nm band-pass emission filters, respectively.

### Correlation analysis at the line level

To integrate the phenotypic measurements obtained across the transgenic lines, eleven variables were assembled at the line level (1 value per line; 8 transgenic lines and the wild type, n = 9): transgene expression, weeks from transplanting to first anthesis, days from anthesis to first ripe fruit, net photosynthesis, total fruit weight per plant, aerial biomass, soluble solids content, fruit volume, stem dry weight, root dry weight, and total GS activity. Pairwise Spearman rank correlation coefficients were computed using complete pairs (n = 7 for comparisons involving fruit volume; n = 9 elsewhere). Pearson coefficients were calculated in parallel and yielded coherent patterns. All analyses were performed in R using base cor.test, with visualization via pheatmap.

### Accession numbers

Sequence data for GS1 and GS2 genes can be found in the solcyc.solgenomics database under the following numbers: *GS1* (*Solyc04g014510*) and *GS2* (*Solyc01g080280*).

## Supplementary Material

koag181_Supplementary_Data

## Data Availability

All data are incorporated into the article and its online supplementary material.

## References

[koag181-B1] Aliyev JA . 2012. Photosynthesis, photorespiration and productivity of wheat and soybean genotypes. Physiol Plant. 145:369–383. 10.1111/j.1399-3054.2012.01613.x. 22420741

[koag181-B2] Alseekh S et al 2021. Mass spectrometry-based metabolomics: a guide for annotation, quantification and best reporting practices. Nat Methods. 18:747–756. 10.1038/s41592-021-01197-1. 34239102 PMC8592384

[koag181-B3] Amthor JS et al 2019. Engineering strategies to boost crop productivity by cutting respiratory carbon loss. Plant Cell. 31:297–314. 10.1105/tpc.18.00743. 30670486 PMC6447004

[koag181-B4] Araújo WL et al 2012. Antisense inhibition of the 2-oxoglutarate dehydrogenase complex in tomato demonstrates its importance for plant respiration and during leaf senescence and fruit maturation. Plant Cell. 24:2328–2351. 10.1105/tpc.112.099002. 22751214 PMC3406899

[koag181-B5] Bachmann M et al 1996. Identification of Ser-543 as the major regulatory phosphorylation site in spinach leaf nitrate reductase. Plant Cell. 8:505–517. 10.1105/tpc.8.3.505. 8721752 PMC161116

[koag181-B6] Backhausen JE, Scheibe R. 1999. Adaptation of tobacco plants to elevated CO_2_: inﬂuence of leaf age on changes in physiology, redox states and NADP-malate dehydrogenase activity. J Exp Bot. 50:665–675. 10.1093/jxb/50.334.665.

[koag181-B7] Bauwe H, Hagemann M, Fernie AR. 2010. Photorespiration: players, partners and origin. Trends Plant Sci. 15:330–336. 10.1016/j.tplants.2010.03.006. 20403720

[koag181-B8] Baxter CJ et al 2005. Fruit carbohydrate metabolism in an introgression line of tomato with increased fruit soluble solids. Plant Cell Physiol. 46:425–437. 10.1093/pcp/pci040. 15695458

[koag181-B9] Bernard SM, Habash DZ. 2009. The importance of cytosolic glutamine synthetase in nitrogen assimilation and recycling. New Phytol. 182:608–620. 10.1111/j.1469-8137.2009.02823.x. 19422547

[koag181-B10] Bienz S, Bisegger P, Guggisberg A, Hesse M. 2005. Polyamine alkaloids. Nat Prod Rep. 22:647–658. 10.1039/b413742f. 16193161

[koag181-B11] Bloom AJ, Burger M, Rubio Asensio JS, Cousins AB. 2010. Carbon dioxide enrichment inhibits nitrate assimilation in wheat and Arabidopsis. Science. 328:899–903. 10.1126/science.1186440. 20466933

[koag181-B12] Bohnert HJ, Nelson DE, Jensen RG. 1995. Adaptations to environmental stresses. Plant Cell. 7:1099–1111. 10.2307/3870060. 12242400 PMC160917

[koag181-B13] Campbell WH, Smarrelli J. 1978. Purification and kinetics of higher plant NADH:nitrate reductase. Plant Physiol. 61:611–616. 10.1104/pp.61.4.611. 16660347 PMC1091928

[koag181-B14] Carroll AJ et al 2015. PhenoMeter: a metabolome database search tool using statistical similarity matching of metabolic phenotypes for high-confidence detection of functional links. Front Bioeng Biotechnol. 3:106. 10.3389/fbioe.2015.00106. 26284240 PMC4518198

[koag181-B15] Caspar T, Huber SC, Somerville C. 1985. Alterations in growth, photosynthesis, and respiration in a starchless mutant of Arabidopsis thaliana(L.) deficient in chloroplast phosphoglucomutase activity. Plant Physiol. 79:11–17. 10.1104/pp.79.1.11. 16664354 PMC1074821

[koag181-B16] Centeno DC et al 2011. Malate plays a crucial role in starch metabolism, ripening, and soluble solid content of tomato fruit and affects postharvest softening. Plant Cell. 23:162–184. 10.1105/tpc.109.072231. 21239646 PMC3051241

[koag181-B17] Chaouch S et al 2010. Peroxisomal hydrogen peroxide is coupled to biotic defense responses by ISOCHORISMATE SYNTHASE1 in a daylength-related manner. Plant Physiol. 153:1692–1705. 10.1104/pp.110.153957. 20543092 PMC2923881

[koag181-B18] Cheung CY, Poolman MG, Fell DA, Ratcliffe RG, Sweetlove LJ. 2014. A diel flux balance model captures interactions between light and dark metabolism during day-night cycles in C3 and crassulacean acid metabolism leaves. Plant Physiol. 165:917–929. 10.1104/pp.113.234468. 24596328 PMC4044858

[koag181-B19] Clark TJ, Guo L, Morgan J, Schwender J. 2020. Modeling plant metabolism: from network reconstruction to mechanistic models. Annu Rev Plant Biol. 71:303–326. 10.1146/annurev-arplant-050718-100221. 32017600

[koag181-B20] Colombié S et al 2015. Modelling central metabolic fluxes by constraint-based optimization reveals metabolic reprogramming of developing *Solanum lycopersicum* (tomato) fruit. Plant J. 81:24–39. 10.1111/tpj.12685. 25279440 PMC4309433

[koag181-B21] de Abreu ELF et al 2017. Metabolic robustness in young roots underpins a predictive model of maize hybrid performance in the field. Plant J. 90:319–329. 10.1111/tpj.13495. 28122143

[koag181-B22] diCenzo GC, Tesi M, Pfau T, Mengoni A, Fondi M. 2020. Genome-scale metabolic reconstruction of the symbiosis between a leguminous plant and a nitrogen-fixing bacterium. PhenoMeter: a metabolome database search tool using statistical similarity matching of metabolic phenotypes for high-confidence detection of functional links Nat Commun. 11:2574. 10.1038/s41467-020-16484-2. PMC7244743 32444627

[koag181-B23] Ding S et al 2023. Phytosulfokine peptide optimizes plant growth and defense via glutamine synthetase GS2 phosphorylation in tomato. EMBO J. 42:e111858. 10.15252/embj.2022111858. 36562188 PMC10015362

[koag181-B24] Do PT, Prudent M, Sulpice R, Causse M, Fernie AR. 2010. The influence of fruit load on the tomato pericarp metabolome in a *Solanum chmielewskii* introgression line population. Plant Physiol. 154:1128–1142. 10.1104/pp.110.163030. 20841452 PMC2971594

[koag181-B25] Dominguez PG et al 2013. ASR1 mediates glucose-hormone cross talk by affecting sugar trafficking in tobacco plants. Plant Physiol. 161:1486–1500. 10.1104/pp.112.208199. 23302128 PMC3585611

[koag181-B26] Ebrahim A, Lerman JA, Palsson BO, Hyduke DR. 2013. COBRApy: constraints-based reconstruction and analysis for python. BMC Syst Biol. 7:74. 10.1186/1752-0509-7-74. 23927696 PMC3751080

[koag181-B27] Eisenhut M et al 2013. Arabidopsis A BOUT DE SOUFFLE is a putative mitochondrial transporter involved in photorespiratory metabolism and is required for meristem growth at ambient CO_2_ levels. Plant J. 73:836–849. 10.1111/tpj.12082. 23181524

[koag181-B28] Fernie AR, Roessner U, Trethewey RN, Willmitzer L. 2001. The contribution of plastidial phosphoglucomutase to the control of starch synthesis within the potato tuber. Planta. 213:418–426. 10.1007/s004250100521. 11506365

[koag181-B29] Fernie AR, Trethewey RN, Krotzky AJ, Willmitzer L. 2004. Metabolite profiling: from diagnostics to systems biology. Nat Rev Mol Cell Biol. 5:763–769. 10.1038/nrm1451. 15340383

[koag181-B30] Fernie AR et al 2011. Recommendations for reporting metabolite data. Plant Cell. 23:2477–2482. 10.1105/tpc.111.086272. 21771932 PMC3226225

[koag181-B31] Fernie AR et al 2013. Perspectives on plant photorespiratory metabolism. Plant Biol. 15:748–753. 10.1111/j.1438-8677.2012.00693.x. 23231538

[koag181-B32] Fernie AR et al 2020. Synchronization of developmental, molecular and metabolic aspects of source-sink interactions. Nat Plants. 6:55–66. 10.1038/s41477-020-0590-x. 32042154

[koag181-B33] Fernie AR, Yan J. 2020. Targeting key genes to tailor old and new crops for a greener agriculture. Mol Plant. 13:354–356. 10.1016/j.molp.2020.02.007. 32074483

[koag181-B34] Foyer CH, Bloom AJ, Queval G, Noctor G. 2009. Photorespiratory metabolism: genes, mutants, energetics, and redox signaling. Ann Rev Plant Biol. 60:455–484. 10.1146/annurev.arplant.043008.091948. 19575589

[koag181-B35] Fridman E, Carrari F, Liu YS, Fernie AR, Zamir D. 2004. Zooming in on a quantitative trait for tomato yield using interspecific introgressions. Science. 305:1786–1789. 10.1126/science.1101666. 15375271

[koag181-B36] Fromm S, Göing J, Lorenz C, Peterhänsel C, Braun HP. 2016. Depletion of the “gamma-type carbonic anhydrase-like” subunits of complex I affects central mitochondrial metabolism in Arabidopsis thaliana. Biochim Biophys Acta. 1857:60–71. 10.1016/j.bbabio.2015.10.006. 26482706

[koag181-B37] Geigenberger P, Fernie AR, Gibon Y, Christ M, Stitt M. 2000. Metabolic activity decreases as an adaptive response to low internal oxygen in growing potato tubers. Biol Chem. 381:723–740. 10.1515/BC.2000.093. 11030430

[koag181-B38] Gibon Y et al 2004. A robot-based platform to measure multiple enzyme activities in Arabidopsis using a set of cycling assays: comparison of changes of enzyme activities and transcript levels during diurnal cycles and in prolonged darkness. Plant Cell. 16:3304–3325. 10.1105/tpc.104.025973. 15548738 PMC535875

[koag181-B39] Grafahrend-Belau E et al 2013. Multiscale metabolic modeling: dynamic flux balance analysis on a whole-plant scale. Plant Physiol. 163:637–647. 10.1104/pp.113.224006. 23926077 PMC3793045

[koag181-B40] Groat RG, Vance CP. 1981. Root nodule enzymes of ammonia assimilation in alfalfa (*Medicago sativa* L.): developmental patterns and response to applied nitrogen. Plant Physiol. 67:1198–1203. 10.1104/pp.67.6.1198. 16661836 PMC425861

[koag181-B41] Hanson KR, Mchale NA. 1988. A starchless mutant of Nicotiana sylvestris containing a modified plastid phosphoglucomutase. Plant Physiol. 88:838–844. 10.1104/pp.88.3.838. 16666394 PMC1055671

[koag181-B42] Heldt HW, Flugge UI. 1987. Subcellular transport of metabolites in a plant cell. In: Stumpf PK, Conn EE, editors. Biochemistry of plants. Academic Press Incorporated, p. 49–85.

[koag181-B150] Hendriks JH et al 2003. ADP-glucose pyrophosphorylase is activated by posttranslational redox-modification in response to light and to sugars in leaves of Arabidopsis and other plant species. Plant Physiol 133:838–49. 12972664 10.1104/pp.103.024513 PMC219057

[koag181-B43] Hodges M . 2002. Enzyme redundancy and the importance of 2-oxoglutarate in plant ammonium assimilation. J Exp Bot. 53:905–916. 10.1093/jexbot/53.370.905. 11912233

[koag181-B44] Hooper CM, Castleden IR, Aryamanesh N, Jacoby RP, Millar AH. 2016. Finding the subcellular location of barley, wheat, rice and maize proteins: the compendium of crop proteins with annotated locations (cropPAL). Plant Cell Physiol. 57:e9. 10.1093/pcp/pcv170. 26556651

[koag181-B45] Hooper CM, Castleden IR, Tanz SK, Aryamanesh N, Millar AH. 2017. SUBA4: the interactive data analysis center for Arabidopsis subcellular protein locations. Nucleic Acids Res. 45:D1064–D1074. 10.1093/nar/gkw1041. 27899614 PMC5210537

[koag181-B46] Hsieh PH et al 2021. The rice PALE1 homolog is involved in the biosynthesis of vitamin B1. Plant Biotechnol J. 19:218–220. 10.1111/pbi.13465. 32777168 PMC7868968

[koag181-B47] Hu M et al 2018. Transgenic expression of plastidic glutamine synthetase increases nitrogen uptake and yield in wheat. Plant Biotechnol J. 16:1858–1867. 10.1111/pbi.12921. 29577547 PMC6181211

[koag181-B48] Kerbler SM, Taylor NL. 2017. Isolation of mitochondria from model and crop plants. Methods Mol Biol. 1670:115–124. 10.1007/978-1-4939-7292-0_12. 28871540

[koag181-B49] Klee HJ, Giovannoni JJ. 2011. Genetics and control of tomato fruit ripening and quality attributes. Annu Rev Genet. 45:41–59. 10.1146/annurev-genet-110410-132507. 22060040

[koag181-B50] Kruger NJ, Ratcliffe RG. 2015. Fluxes through plant metabolic networks: measurements, predictions, insights and challenges. Biochem J. 465:27–38. 10.1042/BJ20140984. 25631681

[koag181-B51] Küken A, Nikoloski Z. 2019. Computational approaches to design and test plant synthetic metabolic pathways. Plant Physiol. 179:894–906. 10.1104/pp.18.01273. 30647083 PMC6393797

[koag181-B52] Kwon CT et al 2020. Rapid customization of Solanaceae fruit crops for urban agriculture. Nat Biotechnol. 38:182–188. 10.1038/s41587-019-0361-2. 31873217

[koag181-B53] Lillo C, Ruoff P. 1992. Hysteretic behavior of nitrate reductase. Evidence of an allosteric binding site for reduced pyridine nucleotides. J Biol Chem. 267:13456–13459. 10.1016/S0021-9258(18)42233-8. 1618848

[koag181-B54] Lin TP, Caspar T, Somerville C, Preiss J. 1988. Isolation and characterization of a starchless mutant of Arabidopsis thaliana(L.) Heynh lacking ADPglucose pyrophosphorylase activity. Plant Physiol. 86:1131–1135. 10.1104/pp.86.4.1131. 16666044 PMC1054640

[koag181-B55] Lisec J, Schauer N, Kopka J, Willmitzer L, Fernie AR. 2006. Gas chromatography mass spectrometry–based metabolite profiling in plants. Nat Protoc. 1:387–396. 10.1038/nprot.2006.59. 17406261

[koag181-B56] Liu X, Hu B, Chu C. 2022. Nitrogen assimilation in plants: current status and future prospects. J Genet Genomics. 49:394–404. 10.1016/j.jgg.2021.12.006. 34973427

[koag181-B57] Lu M-Z, Snyder R, Grant J, Tegeder M. 2020. Manipulation of sucrose phloem and embryo loading affects pea leaf metabolism, carbon and nitrogen partitioning to sinks as well as seed storage pools. Plant J. 101:217–236. 10.1111/tpj.14533. 31520495

[koag181-B58] Lytovchenko A, Bieberich K, Willmitzer L, Fernie AR. 2002. Carbon assimilation and metabolism in potato leaves deficient in plastidial phosphoglucomutase. Planta. 215:802–811. 10.1007/s00425-002-0810-9. 12244446

[koag181-B59] Lytovchenko A et al 2011. Tomato fruit photosynthesis is seemingly unimportant in primary metabolism and ripening but plays a considerable role in seed development. Plant Physiol. 157:1650–1663. 10.1104/pp.111.186874. 21972266 PMC3327185

[koag181-B60] Machado AT, Sodek L, Fernandes MS. 2001. N-partitioning, nitrate reductase and glutamine synthetase activities in two contrasting varieties of maize. Pesq Agropec Bras. 36:249–256. 10.1590/S0100-204X2001000200006.

[koag181-B61] MacRae E, Lunn JE. 2006. Control of sucrose biosynthesis. In: Plaxton WC, McManus MT, editors. Advances in plant research. Vol. 22, Control of primarymetabolism in plants. Blackwell. p. 234–257.

[koag181-B62] Mangel N et al 2019. Enhancement of vitamin B(6) levels in rice expressing Arabidopsis vitamin B(6) biosynthesis de novo genes. Plant J. 99:1047–1065. 10.1111/tpj.14379. 31063672 PMC6852651

[koag181-B63] Martín-Pizarro C et al 2021. The NAC transcription factor FaRIF controls fruit ripening in strawberry. Plant Cell. 33:1574–1593. 10.1093/plcell/koab070. 33624824 PMC8254488

[koag181-B64] Mattoo AK, Handa AK. 2008. Higher polyamines restore and enhance metabolic memory in ripening fruit. Plant Sci. 174:386–393. 10.1016/j.plantsci.2008.01.011.

[koag181-B65] Ménard G, Biais B, Prodhomme D, Ballias P, Gibon Y. 2014. Analysis of enzyme activities. In: Dieuaide-Noubhani M, Alonso AP, editors. Plant metabolic flux analysis: methods and protocols. Humana Press. p. 249–259. 10.1007/978-1-62703-688-7_15 24222420

[koag181-B66] Miflin BJ, Habash DZ. 2002. The role of glutamine synthetase and glutamate dehydrogenase in nitrogen assimilation and possibilities for improvement in the nitrogen utilization of crops. J Exp Bot. 53:979–987. 10.1093/jexbot/53.370.979. 11912240

[koag181-B67] Nashilevitz S et al 2010. An orange ripening mutant links plastid NAD(P)H dehydrogenase complex activity to central and specialized metabolism during tomato fruit maturation. Plant Cell. 22:1977–1997. 10.1105/tpc.110.074716. 20571113 PMC2910969

[koag181-B68] Nelson BK, Cai X, Nebenführ A. 2007. A multicolored set of in vivo organelle markers for co-localization studies in Arabidopsis and other plants. Plant J. 51:1126–1136. 10.1111/j.1365-313X.2007.03212.x. 17666025

[koag181-B69] Noguchi K, Yoshida K. 2008. Interaction between photosynthesis and respiration in illuminated leaves. Mitochondrion. 8:87–99. 10.1016/j.mito.2007.09.003. 18024239

[koag181-B70] Nunes-Nesi A et al 2005. Enhanced photosynthetic performance and growth as a consequence of decreasing mitochondrial malate dehydrogenase activity in transgenic tomato plants. Plant Physiol. 137:611–622. 10.1104/pp.104.055566. 15665243 PMC1065362

[koag181-B71] Nunes-Nesi A, Fernie AR, Stitt M. 2010. Metabolic and signaling aspects underpinning the regulation of plant carbon nitrogen interactions. Mol Plant. 3:973–996. 10.1093/mp/ssq049. 20926550

[koag181-B72] Obiadalla-Ali H, Fernie AR, Lytovchenko A, Kossmann J, Lloyd JR. 2004. Inhibition of chloroplastic fructose 1,6-bisphosphatase in tomato fruits leads to decreased fruit size, but only small changes in carbohydrate metabolism. Planta. 219:533–540. 10.1007/s00425-004-1257-y. 15060828

[koag181-B73] Omena-Garcia RP, Araujo WL, Gibon Y, Fernie AR, Nunes-Nesi A. 2017. Measurement of tricarboxylic acid cycle enzyme activities in plants. Methods Mol Biol. 1670:167–182. 10.1007/978-1-4939-7292-0_14. 28871542

[koag181-B74] Osorio S et al 2013. Alteration of the interconversion of pyruvate and malate in the plastid or cytosol of ripening tomato fruit invokes diverse consequences on sugar but similar effects on cellular organic acid, metabolism, and transitory starch accumulation. Plant Physiol. 161:628–643. 10.1104/pp.112.211094. 23250627 PMC3561009

[koag181-B75] Perchlik M, Tegeder M. 2018. Leaf amino acid supply affects photosynthetic and plant nitrogen use efficiency under nitrogen stress. Plant Physiol. 178:174–188. 10.1104/pp.18.00597. 30082496 PMC6130036

[koag181-B151] Porra RJ Scheer H. 2009. Towards a more accurate future for chlorophyll a and b determinations: the inaccuracies of Daniel Arnon's assay. Photosynth Res. 140:215–219. 10.1007/s11120-018-0579-8 30194670

[koag181-B76] Rademacher T et al 2002. An engineered phosphoenolpyruvate carboxylase redirects carbon and nitrogen flow in transgenic potato plants. Plant J. 32:25–39. 10.1046/j.1365-313X.2002.01397.x. 12366798

[koag181-B77] Rastogi R, Davies PJ. 1991. Polyamine metabolism in ripening tomato fruit: II. polyamine metabolism and synthesis in relation to enhanced putrescine content and storage life of a/c tomato fruit. Plant Physiol. 95:41–45. 10.1104/pp.95.1.41. 16667978 PMC1077482

[koag181-B78] Ren Y et al 2021. Evolutionary gain of oligosaccharide hydrolysis and sugar transport enhanced carbohydrate partitioning in sweet watermelon fruits. Plant Cell. 33:1554–1573. 10.1093/plcell/koab055. 33570606 PMC8254481

[koag181-B79] Riedelsheimer C et al 2012a. Genomic and metabolic prediction of complex heterotic traits in hybrid maize. Nat Genet. 44:217–220. 10.1038/ng.1033. 22246502

[koag181-B80] Riedelsheimer C et al 2012b. Genome-wide association mapping of leaf metabolic profiles for dissecting complex traits in maize. Proc Nat Acad Sci U S A. 109:8872–8877. 10.1073/pnas.1120813109. PMC3384205 22615396

[koag181-B81] Robaina-Estévez S, Daloso DM, Zhang Y, Fernie AR, Nikoloski Z. 2017. Resolving the central metabolism of Arabidopsis guard cells. Sci Rep. 7:8307. 10.1038/s41598-017-07132-9. 28814793 PMC5559522

[koag181-B82] Robinson SA et al 1991. The role of glutamate dehydrogenase in plant nitrogen metabolism. Plant Physiol. 95:509–516. 10.1104/pp.95.2.509. 16668014 PMC1077561

[koag181-B83] Sánchez-Sevilla JF et al 2017. Gene expression atlas of fruit ripening and transcriptome assembly from RNA-seq data in octoploid strawberry (*Fragaria×ananassa*). Sci Rep. 7:13737. 10.1038/s41598-017-14239-6. 29062051 PMC5653846

[koag181-B84] Santiago JP, Tegeder M. 2016. Connecting source with sink: the role of Arabidopsis AAP8 in phloem loading of amino acids. Plant Physiol. 171:508–521. 10.1104/pp.16.00244. 27016446 PMC4854717

[koag181-B85] Santiago JP, Tegeder M. 2017. Implications of nitrogen phloem loading for carbon metabolism and transport during Arabidopsis development. J Integr Plant Biol. 59:409–421. 10.1111/jipb.12533. 28296149

[koag181-B86] Schauer N et al 2006. Comprehensive metabolic profiling and phenotyping of interspecific introgression lines for tomato improvement. Nat Biotechnol. 24:447–454. 10.1038/nbt1192. 16531992

[koag181-B87] Schauer N et al 2008. Mode of inheritance of primary metabolic traits in tomato. Plant Cell. 20:509–523. 10.1105/tpc.107.056523. 18364465 PMC2329927

[koag181-B88] Scheibe R . 1991. Redox-modulation of chloroplast enzymes: a common principle for individual control. Plant Physiol. 96:1–3. 10.1104/pp.96.1.1. 16668135 PMC1080704

[koag181-B89] Scheibe R, Backhausen JE, Emmerlich V, Holtgrefe S. 2005. Strategies to maintain redox homeostasis during photosynthesis under changing conditions. J Exp Bot. 56:1481–1489. 10.1093/jxb/eri181. 15851411

[koag181-B90] Schippers JH et al 2008. The Arabidopsis onset of leaf death5 mutation of quinolinate synthase affects nicotinamide adenine dinucleotide biosynthesis and causes early ageing. Plant Cell. 20:2909–2925. 10.1105/tpc.107.056341. 18978034 PMC2590718

[koag181-B91] Seger M, Gebril S, Tabilona J, Peel A, Sengupta-Gopalan C. 2015. Impact of concurrent overexpression of cytosolic glutamine synthetase (GS1) and sucrose phosphate synthase (SPS) on growth and development in transgenic tobacco. Planta. 241:69–81. 10.1007/s00425-014-2165-4. 25213117

[koag181-B92] Seymour GB, Østergaard L, Chapman NH, Knapp S, Martin C. 2013. Fruit development and ripening. Annu Rev Plant Biol. 64:219–241. 10.1146/annurev-arplant-050312-120057. 23394500

[koag181-B93] Shameer S, Ratcliffe RG, Sweetlove LJ. 2019. Leaf energy balance requires mitochondrial respiration and export of chloroplast NADPH in the light. Plant Physiol. 180:1947–1961. 10.1104/pp.19.00624. 31213510 PMC6670072

[koag181-B94] Shameer S, Vallarino JG, Fernie AR, Ratcliffe RG, Sweetlove LJ. 2020. Flux balance analysis of metabolism during growth by osmotic cell expansion and its application to tomato fruits. Plant J. 103:68–82. 10.1111/tpj.14707. 31985867

[koag181-B95] Sonnewald U et al 1997. Increased potato tuber size resulting from apoplastic expression of a yeast invertase. Nat Biotechnol. 15:794–797. 10.1038/nbt0897-794. 9255797

[koag181-B96] Sonnewald U, Fernie AR. 2018. Next-generation strategies for understanding and influencing source-sink relations in crop plants. Curr Opin Plant Biol. 43:63–70. 10.1016/j.pbi.2018.01.004. 29428477

[koag181-B97] Sonnewald U et al 2020. The cassava source-sink project: opportunities and challenges for crop improvement by metabolic engineering. Plant J. 103:1655–1665. 10.1111/tpj.14865. 32502321

[koag181-B98] Stitt M . 1990a. Fructose-2,6-bisphosphate as a regulatory molecule in plants. Annu Rev Plant Physiol Plant Mol Biol. 41:153–185. 10.1146/annurev.pp.41.060190.001101.

[koag181-B99] Stitt M . 1990b. Fructose-2,6-bisphosphate. In: Methods in plant biochemistry. Academic Press Incorporated. p. 87–93.

[koag181-B100] Sweetlove LJ, Muller-Rober B, Willmitzer L, Hill SA. 1999. The contribution of adenosine 5′-diphosphoglucose pyrophosphorylase to the control of starch synthesis in potato tubers. Planta. 209:330–337. 10.1007/s004250050640. 10502100

[koag181-B101] Sweetlove LJ, Nielsen J, Fernie AR. 2017. Engineering central metabolism—a grand challenge for plant biologists. Plant J. 90:749–763. 10.1111/tpj.13464. 28004455

[koag181-B102] Szecowka M et al 2012. Decreasing the mitochondrial synthesis of malate in potato tubers does not affect plastidial starch synthesis, suggesting that the physiological regulation of ADPglucose pyrophosphorylase is context dependent. Plant Physiol. 160:2227–2238. 10.1104/pp.112.204826. 23064409 PMC3510143

[koag181-B103] Taira M, Valtersson I, Burkhardt B, Ludwig R. 2004. Arabidopsis thaliana GLN2-enconded glutamine synthetase is dual targeted to leaf mitochondria and chloroplasts. Plant Cell. 16:2048–2058. 10.1105/tpc.104.022046. 15273293 PMC519197

[koag181-B104] Tan XLJ, Cheung CYM. 2020. A multiphase flux balance model reveals flexibility of central carbon metabolism in guard cells of C3 plants. Plant J. 104:1648–1656. 10.1111/tpj.15027. 33070390

[koag181-B105] Tegeder M, Masclaux-Daubresse C. 2018. Source and sink mechanisms of nitrogen transport and use. New Phytol. 217:35–53. 10.1111/nph.14876. 29120059

[koag181-B106] Tezara W, Martínez D, Rengifo E, Herrera A. 2003. Photosynthetic responses of the tropical spiny shrub Lycium nodosum (Solanaceae) to drought, soil salinity and saline spray. Ann Bot. 92:757–765. 10.1093/aob/mcg199. 14534200 PMC4243616

[koag181-B107] Thomsen HC, Eriksson D, Møller IS, Schjoerring JK. 2014. Cytosolic glutamine synthetase: a target for improvement of crop nitrogen use efficiency? Trends Plant Sci. 19:656–663. 10.1016/j.tplants.2014.06.002. 25017701

[koag181-B108] Timm S . 2020. The impact of photorespiration on plant primary metabolism through metabolic and redox regulation. Biochem Soc Trans. 48:2495–2504. 10.1042/BST20200055. 33300978

[koag181-B109] Töpfer N, Braam T, Shameer S, Ratcliffe RG, Sweetlove LJ. 2020. Alternative crassulacean acid metabolism modes provide environment-specific water-saving benefits in a leaf metabolic model. Plant Cell. 32:3689–3705. 10.1105/tpc.20.00132. 33093147 PMC7721317

[koag181-B110] Vallarino JG et al 2020. Multi-gene metabolic engineering of tomato plants results in increased fruit yield up to 23%. Sci Rep. 10:17219. 10.1038/s41598-020-73709-6. 33057137 PMC7560729

[koag181-B111] Valle EM, Boggio SB, Heldt HW. 1998. Free amino acid composition of phloem sap and growing fruit of *Lycopersicon esculentum*. Plant Cell Physiol. 39:458–461. 10.1093/oxfordjournals.pcp.a029391.

[koag181-B112] Vera-Vives AM et al 2025. Cytochrome c oxidase inactivation in Physcomitrium patens reveals that respiration coordinates plant metabolism. Plant Cell. 37:koaf101. 10.1093/plcell/koaf101. 40324757 PMC12164586

[koag181-B113] Vincentz M, Moureaux T, Leydecker M-T, Vaucheret H, Caboche M. 1993. Regulation of nitrate and nitrite reductase expression in Nicotiana plumbaginifolia leaves by nitrogen and carbon metabolites. Plant J. 3:315–324. 10.1111/j.1365-313X.1993.tb00183.x. 8220446

[koag181-B114] Von Wettstein D, Gough S, Kannangara CG. 1995. Chlorophyll biosynthesis. Plant Cell. 7:1039–1057. 10.2307/3870056. 12242396 PMC160907

[koag181-B115] Walker AJ, Ho LC. 1977. Carbon translocation in the tomato: carbon import and fruit growth1. Ann Bot. 41:813–823. 10.1093/oxfordjournals.aob.a085357.

[koag181-B116] Wang S et al 2008. Altered plastid levels and potential for improved fruit nutrient content by downregulation of the tomato DDB1-interacting protein CUL4. Plant J. 55:89–103. 10.1111/j.1365-313X.2008.03489.x. 18363785

[koag181-B117] Wu D et al 2021. Increased glutamine synthetase by overexpression of TaGS1 improves grain yield and nitrogen use efficiency in rice. Plant Physiol Biochem. 169:259–268. 10.1016/j.plaphy.2021.11.021. 34814097

[koag181-B118] Yuan H, Cheung CY, Poolman MG, Hilbers PA, van Riel NA. 2016. A genome-scale metabolic network reconstruction of tomato (*Solanum lycopersicum* L.) and its application to photorespiratory metabolism. Plant J. 85:289–304. 10.1111/tpj.13075. 26576489

[koag181-B119] Zanor MI et al 2009. RNA interference of LIN5 in tomato confirms its role in controlling brix content, uncovers the influence of sugars on the levels of fruit hormones, and demonstrates the importance of sucrose cleavage for normal fruit development and fertility. Plant Physiol. 150:1204–1218. 10.1104/pp.109.136598. 19439574 PMC2705052

[koag181-B120] Zhang L, Garneau MG, Majumdar R, Grant J, Tegeder M. 2015. Improvement of pea biomass and seed productivity by simultaneous increase of phloem and embryo loading with amino acids. Plant J. 81:134–146. 10.1111/tpj.12716. 25353986

[koag181-B121] Zhang Y et al 2020. A highly efficient agrobacterium-mediated method for transient gene expression and functional studies in multiple plant species. Plant Commun. 1:100028. 10.1016/j.xplc.2020.100028. 33367253 PMC7747990

[koag181-B122] Zhu Q et al 2017. Development of “purple endosperm rice” by engineering anthocyanin biosynthesis in the endosperm with a high-efficiency transgene stacking system. Mol Plant. 10:918–929. 10.1016/j.molp.2017.05.008. 28666688

[koag181-B123] Zrenner R, Salanoubat M, Willmitzer L, Sonnewald U. 1995. Evidence of the crucial role of sucrose synthase for sink strength using transgenic potato plants (*Solanum tuberosum* L.). Plant J. 7:97–107. 10.1046/j.1365-313X.1995.07010097.x. 7894514

