# Supplementary material for: Mitochondrial glutamine synthetase overexpression in tomato source leaves increases assimilate export and fruit yield
Source: Plant Cell. 2026 Jun 13;38(8):koag181. doi: 10.1093/plcell/koag181 (PMC13439946; doi:10.1093/plcell/koag181)
Supplement: Supplementary file 1 [file koag181_supplementary_data.zip › Supple. Table 1.docx]

**Supplemental Table 1. List of constraints applied to the diel stoichiometric model of tomato leaf metabolism.** Abbreviations: NGAM = Non-growth associated maintenance; PPFD = Photosynthetically active photon flux density

| Reaction ID(s) | Name | Value | Comment / Reference |
| --- | --- | --- | --- |
| Sucrose_tx1 | Day-time sucrose uptake | 0 |  |
| GLC_tx1 | Day-time glucose uptake | 0 |  |
| NH4_tx1 | Day-time ammonium uptake | 0 |  |
| Photon_tx1 | Day-time photon uptake | 500 |  |
| CO2_tx1 | Day-time CO_2_ exchange | 0 ≤ *v* ≤ 1000, where *v* is flux |  |
| G6P_Pi_pc1 | Day-time plastidic G6P transporter | 0 | (Lloyd & Zakhleniuk, 2004; Weise et al., 2011) |
| Plastoquinol_Oxidase_p1 | Day-time plastidic terminal oxidase | 0 |  |
| 2PGADEHYDRAT_RXN_p1 | Day-time plastidic enolase | 0 | (Prabhakar et al., 2009) |
| NADPH_Dehydrogenase_p1 | Day-time NADPH dehydrogenase | 0 | (Yamamoto et al., 2011) |
| ATP_ADP_Pi_pc1 | Day-time plastidic nucleotide transporters | 0 | (Voon et al., 2018) |
| ATPase_tx1 | Day-time NGAM ATP hydrolysis flux | 5.2351 | (0.0049×PPFD) + 2.7851 |
| Sucrose_tx2 | Night-time sucrose uptake | 0 |  |
| GLC_tx2 | Night-time glucose uptake | 0 |  |
| NH4_tx2 | Night-time ammonium uptake | 0 |  |
| Photon_tx2 | Night-time photon uptake | 0 |  |
| CO2_tx2 | Night-time CO_2_ exchange | -1000 ≤ *v* ≤ 0, where *v* is flux |  |
| G6P_Pi_pc2 | Night-time plastidic G6P transporter | 0 | (Lloyd & Zakhleniuk, 2004; Weise et al., 2011) |
| 2PGADEHYDRAT_RXN_p2 | Night-time plastidic enolase | 0 | (Prabhakar et al., 2009) |
| NADPH_Dehydrogenase_p2 | Night-time NADPH dehydrogenase | 0 | (Yamamoto et al., 2011) |
| ATP_ADP_Pi_pc2 | Night-time plastidic nucleotide transporters | 0 | (Voon et al., 2018) |
| ATPase_tx2 | Night-time NGAM ATP hydrolysis flux | 5.2351 | (0.0049×PPFD) + 2.7851 |
| $\frac{Phloem\_output\_tx1}{Phloem\_output\_tx2}$ | Day-night phloem export rate ratio | 3 |  |
| $\frac{Nitrate\_ec1}{Nitrate\_ec2}$ | Day-night nitrate uptake rate ratio | 3/2 |  |
| $\frac{\mathrm{RIBULOSE}\_\mathrm{BISPHOSPHATE}\_\mathrm{CARBOXYLASE}\_\mathrm{RXN}\_p1}{\mathrm{RXN}\_961\_p1}$ | Rubisco carboxylase oxygenase ratio | 3 |  |
| $\frac{ATPase\_tx1}{(NADPHoxc\_tx1+NADPHoxp\_tx1 +NADPHoxm\_tx1)}$ | NGAM associated ATP hydrolysis - NADPH oxidase ratio | 3 |  |
| $\frac{ATPase\_tx2}{(NADPHoxc\_tx2+NADPHoxp\_tx2 +NADPHoxm\_tx2)}$ | NGAM associated ATP hydrolysis - NADPH oxidase ratio | 3 |  |

**References:**

**Lloyd, J. C., & Zakhleniuk, O. V.** (2004). Responses of primary and secondary metabolism to sugar accumulation revealed by microarray expression analysis of the Arabidopsis mutant, pho3. J Exp Bot. **55**(400): 1221–1230. https://doi.org/10.1093/jxb/erh143

**Weise, S. E., van Wijk, K. J., and Sharkey, T. D.** (2011). The role of transitory starch in C3, CAM, and C4 metabolism and opportunities for engineering leaf starch accumulation. J Exp Bot. **62**(9): 3109–3118. https://doi.org/10.1093/jxb/err035

**Prabhakar, V., Löttgert, T., Gigolashvili, T., Bell, K., Flügge, U. I., and Häusler, R. E. (2009).** Molecular and functional characterization of the plastid-localized Phosphoenolpyruvate enolase (ENO1) from Arabidopsis thaliana. FEBS Letters. **583**(6): 983–991. https://doi.org/10.1016/j.febslet.2009.02.017

**Yamamoto, H., Peng, L., Fukao, Y., and Shikanai, T. (2011).** An Src homology 3 domain-like fold protein forms a ferredoxin binding site for the chloroplast NADH dehydrogenase-like complex in Arabidopsis.  Plant Cell. **23**(4): 1480–1493. https://doi.org/10.1105/tpc.110.080291

**Voon, C. P., Guan, X., Sun, Y., Sahu, A., Chan, M. N., Gardeström, P., Wagner, S., Fuchs, P., Nietzel, T., Versaw, W. K., Schwarzländer, M., and Lim, B. L. (2018).** ATP compartmentation in plastids and cytosol of Arabidopsis thaliana revealed by fluorescent protein sensing. Proc. Natl. Acad. Sci. USA. **115**(45): E10778–E10787. https://doi.org/10.1073/pnas.1711497115

**Töpfer, N., Braam, T., Shameer, S., Ratcliffe, R. G., and Sweetlove, L. J. (2020).** Alternative CAM Modes Provide Environment-Specific Water-Saving Benefits in a Leaf Metabolic Model. Plant Cell. **32**. tpc.00132.2020. https://doi.org/10.1105/tpc.20.00132
