# Supplementary material for: Mitochondrial glutamine synthetase overexpression in tomato source leaves increases assimilate export and fruit yield
Source: Plant Cell. 2026 Jun 13;38(8):koag181. doi: 10.1093/plcell/koag181 (PMC13439946; doi:10.1093/plcell/koag181)
Supplement: Supplementary file 1 [file koag181_supplementary_data.zip › Supplemental_Figures_v2.pdf]

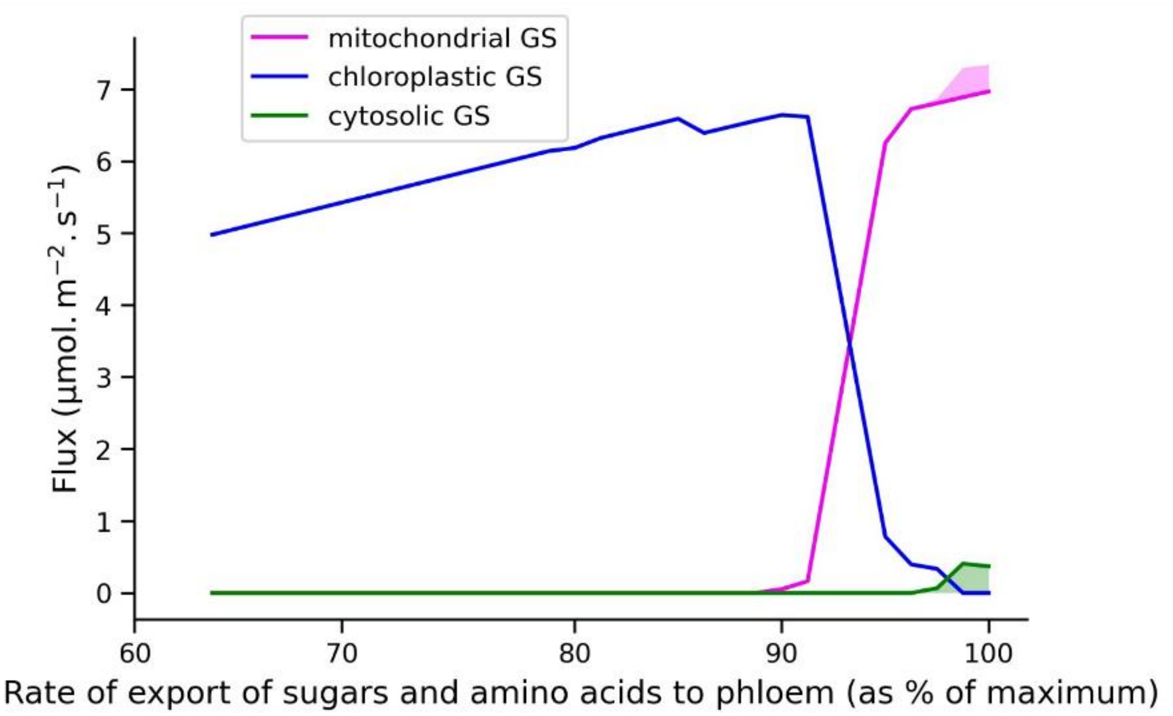

Supplemental Figure 1: Predicted glutamine synthetase fluxes in mature tomato leaves as the export rate of sugar and amino acids to the phloem was varied between 60 – 100% of the maximum achievable within the model constraints. Solid lines represent the flux values in a parsimonious flux balance analysis (pFBA) solution for the specified phloem export rate and shaded regions represent the range of feasible flux values as predicted by flux variability analysis (FVA). Fluxes are for the reactions catalysed by mitochondrial, chloroplastic and cytosolic glutamine synthetases, as indicated.

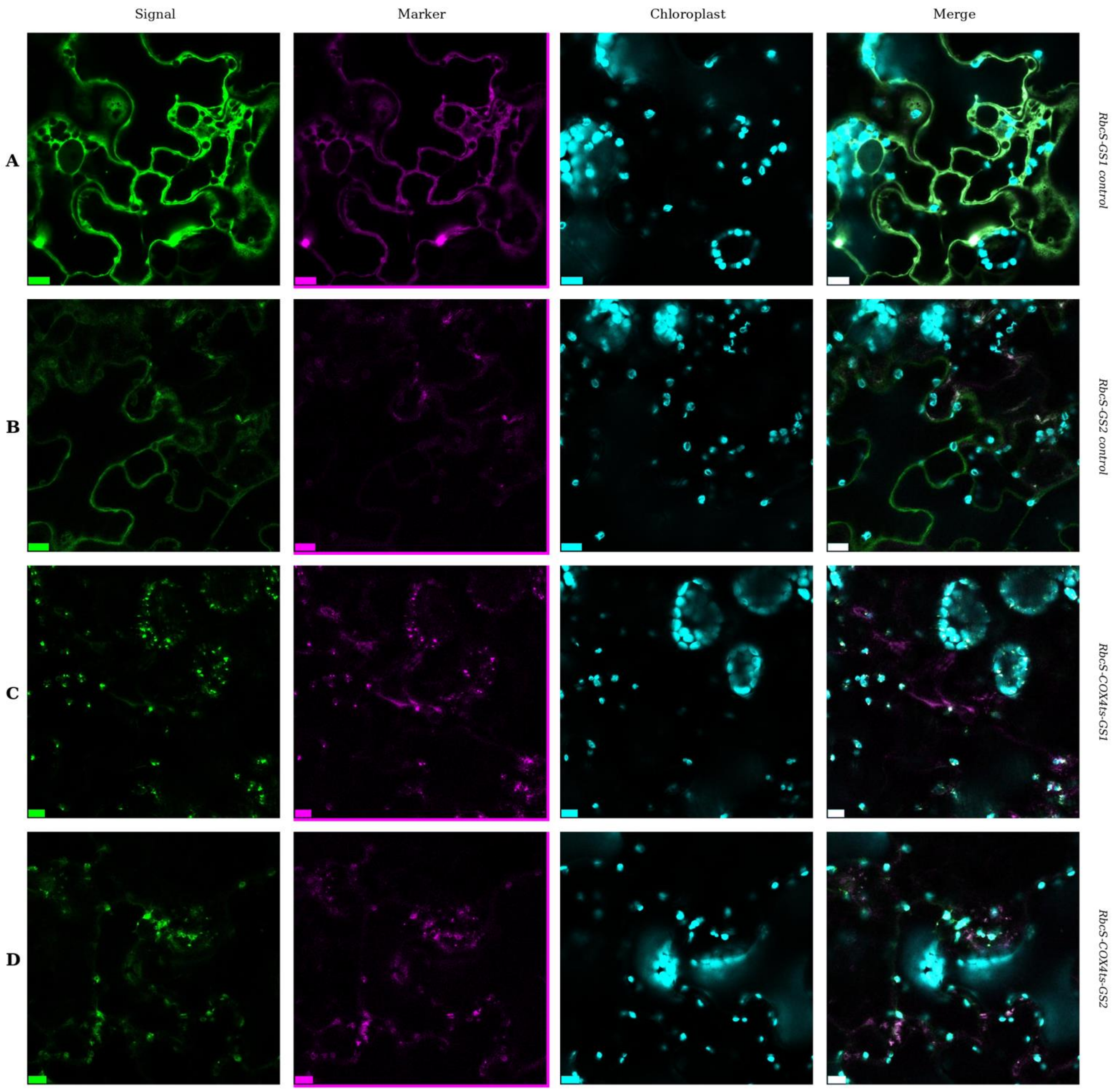

Supplemental Figure 2. Subcellular localization of *SlGS1* and *SlGS2* fusion proteins by transient expression in *N. benthamiana* leaves. GFP-tagged coding sequences were co-expressed with organelle-specific mCherry markers. **(A)** RbcS-GS1 (without COX4ts transit peptide) showing cytosolic GFP signal co-localizing with cytosolic mCherry marker. **(B)** RbcS-GS2 (without COX4ts transit peptide) showing GFP signal co-localizing with plastidial mCherry marker. **(C)** RbcS-COX4ts-GS1 showing GFP signal co-localizing with mitochondrial mCherry marker. **(D)** RbcS-COX4ts-GS2 showing GFP signal co-localizing with mitochondrial mCherry marker. Columns from left to right: GFP signal, mCherry marker, chloroplast autofluorescence, and merged image. Scale bars, 10 µm. The mCherry signal is displayed in magenta to improve accessibility for readers with red–green colour vision deficiency, following editorial guidelines.

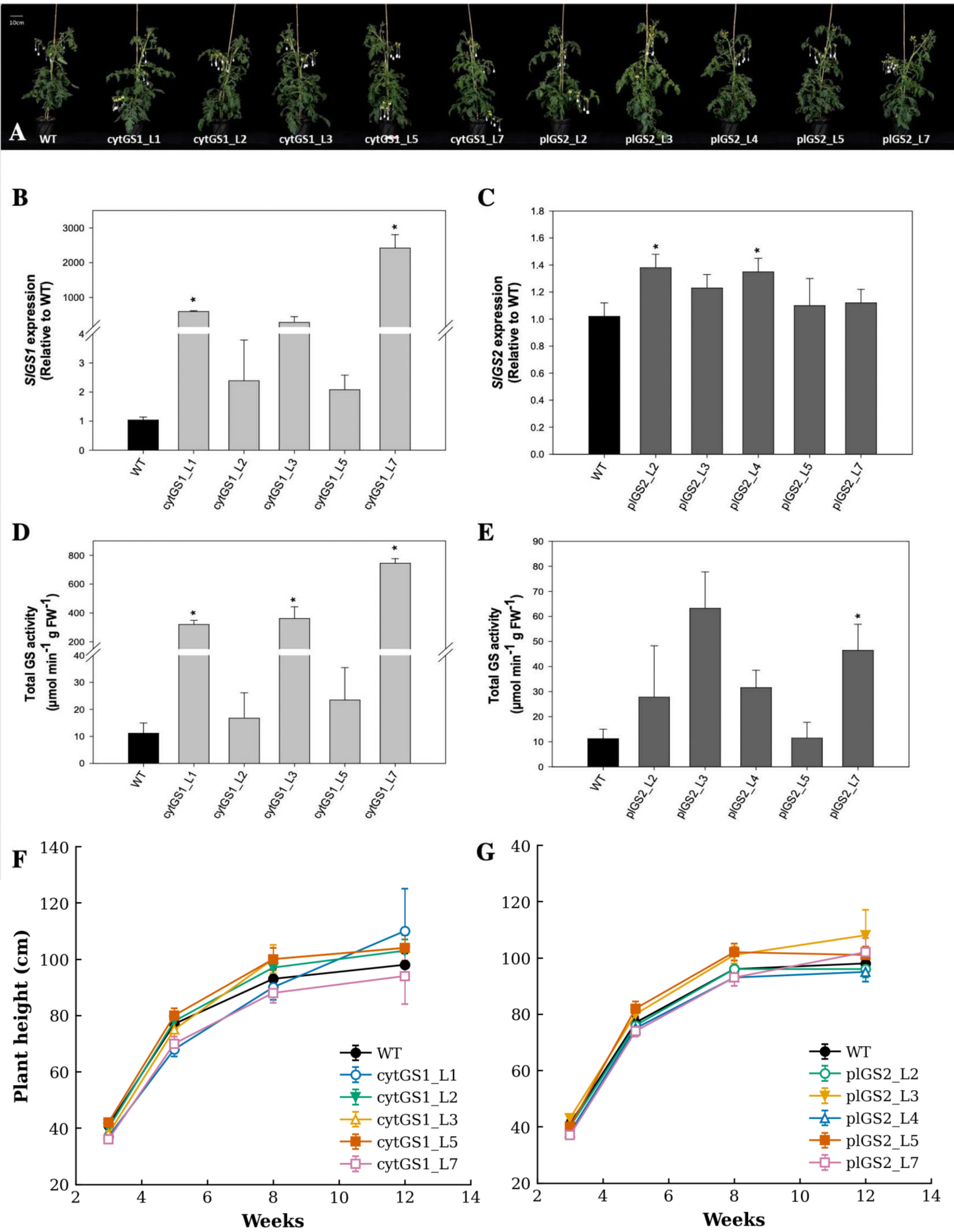

Supplemental Figure 3. Characterization of cytGS1 (RbcS-GS1) and plGS2 (RbcS-GS2). **(A)** Pictures of 5-week old plants. **(B, C)** Expression of tomato *SlGS1* and *SlGS2* genes by qRT-PCR in source leaves. **(D, E)** Total GS activity in source leaves (enzyme activity was normalized to dry weight). **(F, G)** Plant height along plant development. Values are means from 3-5 replicates ± standard error. Statistically significant differences by Student's *t* test are indicated by an asterisk ($P \leq 0.05$).

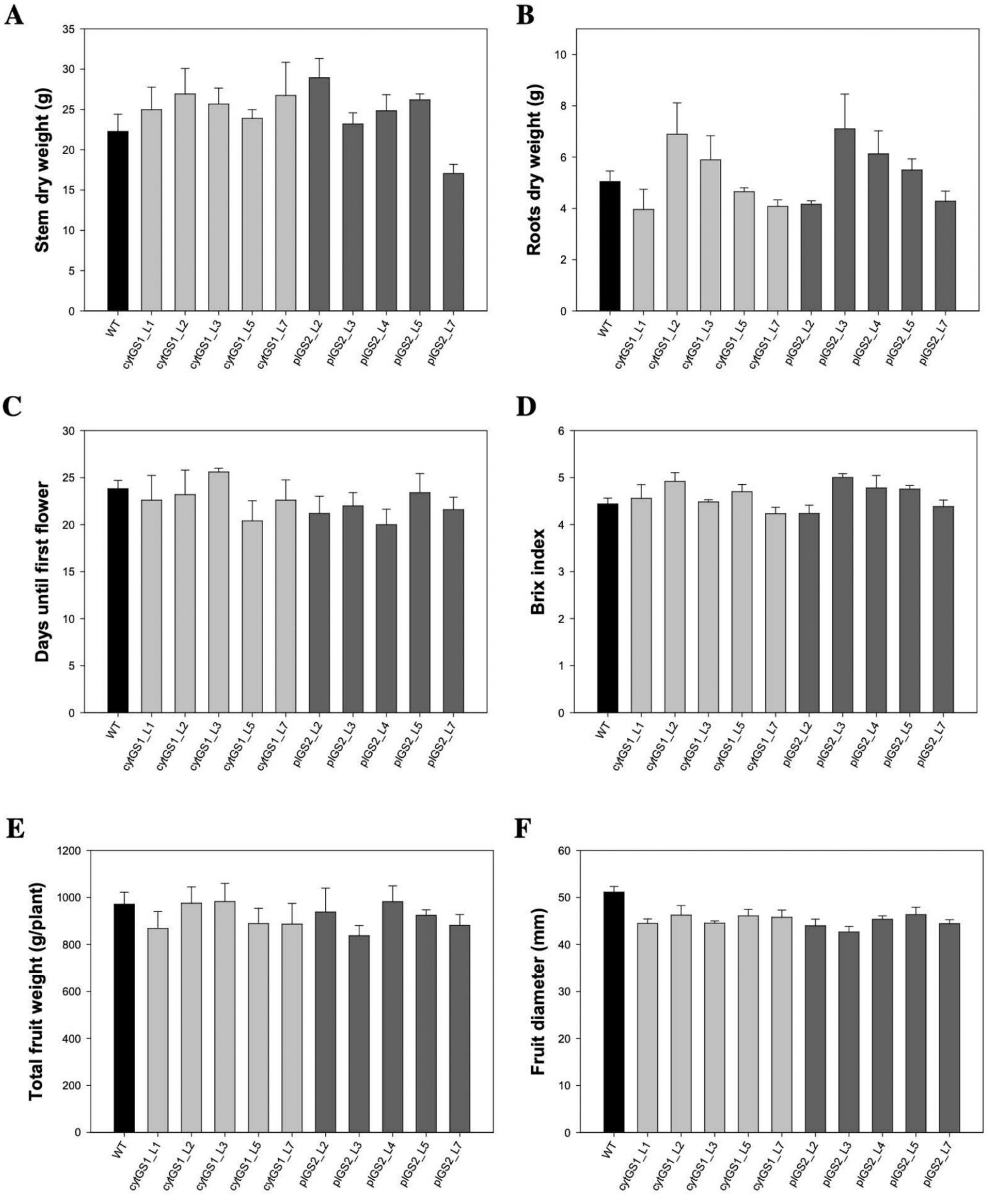

Supplemental Figure 4. Characterization of cytGS1 (RbcS-GS1) and plGS2 (RbcS-GS2). **(A, B)** Total stem and root dry weight in plants at 16 weeks, respectively. (C) Number of days until first flower appears after planting. (D) Soluble solid content (°Brix). (E) Total fruit yield. (F) Fruit diameter. Values are means from 3-5 replicates ± standard error. Statistically significant differences by Student's *t* test are indicated by an asterisk ($P \leq 0.05$).

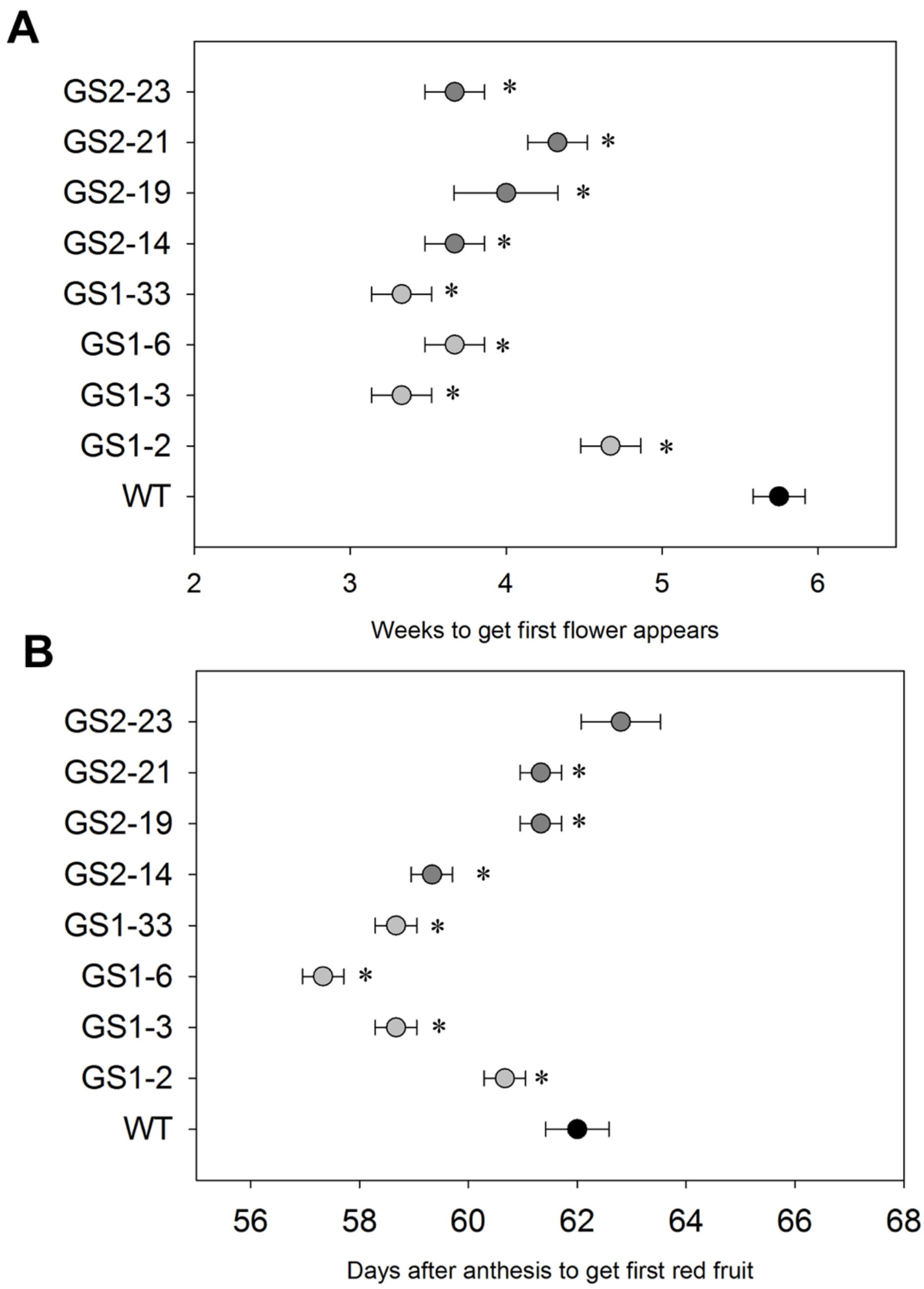

Supplemental Figure 5. Flower and ripe fruit appearance in *RbcS–GS1* and *RbcS–GS2* lines. **(A)** Number of weeks to get first flower appears after planting. **(B)** Number of days after anthesis to get first red fruit. Asterisks denote values that were significant differences by t-tests ($P<0.05$). Values indicate means ± SE from five replicate of each line.

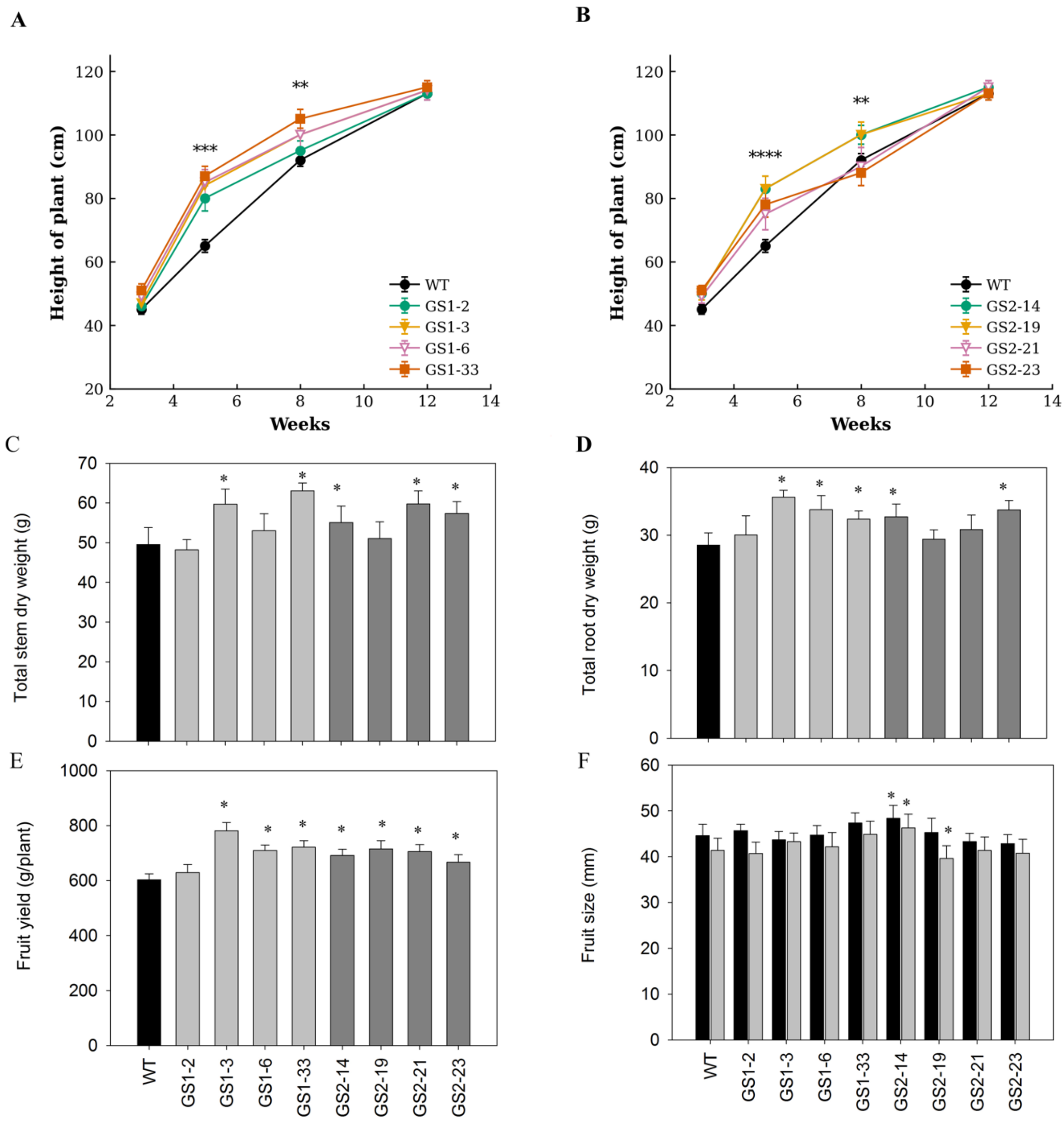

Supplemental Figure 6. Phenotypic characterization of the *RbcS–GS1* and *RbcS–GS2* lines grown in Autumn season. **(A, B)** Plant height along plant development. **(C, D)** Total stem and root dry weight in plants of 16 weeks, respectively. **(E)** Total fruit yield. **(F)** Total fruit size. Values are showed as means ± SE of five individual plants (black and grey bars indicate width and height, respectively). Asterisk indicate statistically significant differences determined by Student ́s *t* test (P<0.05).

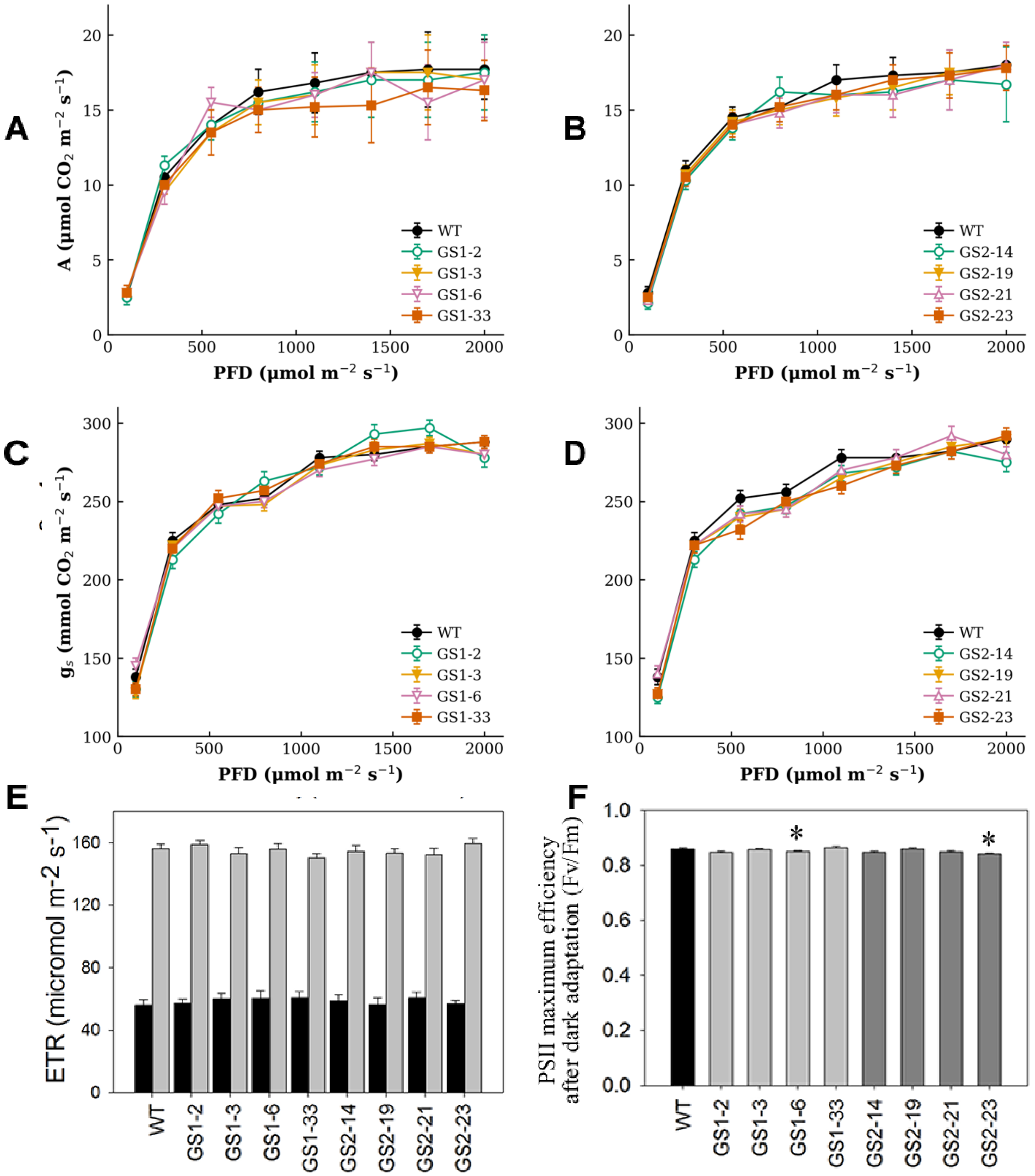

Supplemental Figure 7 Photosynthetic parameters in *RbcS–GS1* and *RbcS–GS2* lines. **(A, B)** Net photosynthesis rate. **(C, D)** Stomata conductance. **(E)** Relative electron transport rates (ETR) at high (700 μmol m^{-2}; grey bars) and low (100 μmol m^{-2}; black bars) irradiances. **(F)** Maximum PSII photochemical efficiency (Fv/Fm).All measurements were performed in six-week old plants. Values are showed as means ± SE of five individual plants. Asterisk indicate statistically significant differences determined by Studentˊs *t* test (*P*<0.05).

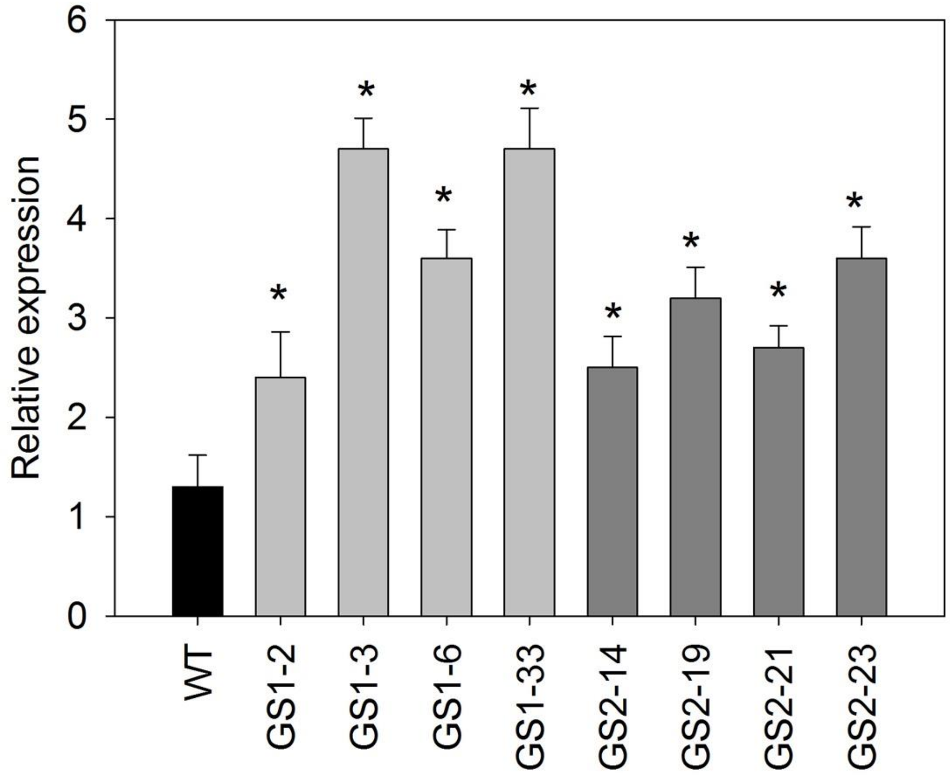

Supplemental Figure 8. Expression of tomato *GDC-L gene* by qRT-PCR. Data were obtained from the third fully expanded leaf from the apex of six-week-old plants. Leaves were harvested at midday. Values are means from five replicates ± SE. Asterisk indicate statistically significant differences determined by Student ́s *t* test ($P<0.05$).

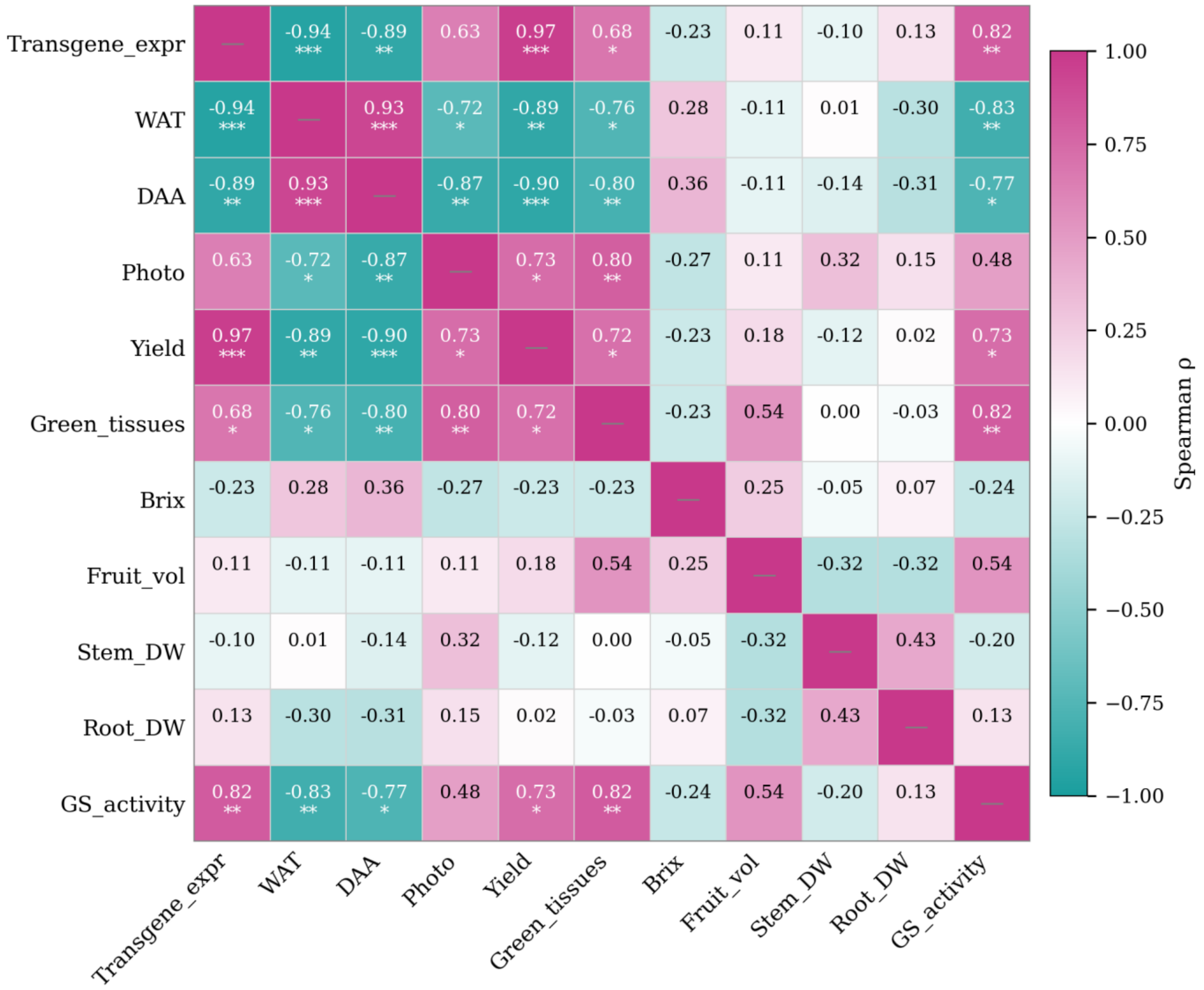

Supplemental Figure 9. Line-level correlation matrix of phenotypic and molecular traits across the transgenic lines and wild-type control (n = 9). Pairwise Spearman rank correlation coefficients (ρ) are shown in each cell for the eleven variables analysed: transgene expression, weeks from transplanting to first anthesis, days from anthesis to first ripe fruit, net photosynthesis, total fruit weight per plant, aerial biomass, soluble solids content, fruit volume, stem dry weight, root dry weight, and total GS activity. n = 7 for comparisons involving fruit volume; n = 9 elsewhere. Cell colour encodes the magnitude and sign of ρ from cyan (negative) to magenta (positive). Asterisks indicate significance: *$P < 0.05$, **$P < 0.01$, ***$P < 0.001$. The diagonal is marked with a dash. See Methods for further details on the correlation analysis.

Enzyme	Compartment	Wild Type	GS1-3	GS2-14
**GS**	Mitochondria	10.5 ± 3.2	41.2 ± 2.5*	29.1 ± 3.1*
**SDH**	Mitochondria	111.6 ± 6.1	140.1 ± 6.7	117.5 ± 7.5
**UGPase**	Cytosol	0.27 ± 0.24	0.36 ± 0.22	0.59 ± 0.23
**AGPase**	Plastid	n.d.	n.d.	n.d.

Values are nmol min^{-1} gFW^{-1} (mean ± SE, n = 5). *P < 0.01 vs wild type (Student's t test).
n.d., not detected (activity below detection limit; measurements included negative values).

Supplemental Table 3. Enzyme activities in purified mitochondrial fractions isolated from leaves of wild type, GS1-3, and GS2-14 plants. Glutamine synthetase (GS), succinate dehydrogenase (SDH, mitochondrial marker), UDP-glucose pyrophosphorylase (UGPase, cytosolic marker), and ADP-glucose pyrophosphorylase (AGPase, plastidial marker). Values are nmol min^{-1} gFW^{-1} (mean ± SE, n = 5). Asterisks indicate significant differences compared to wild type (Student's *t* test, $P < 0.01$). n.d., not detected (activity below detection limit).
